# Seeded‐Growth of ZIF‐8 onto Magnetic Nanoparticles: Smart Nanocarriers with Ultrahigh Drug Loading for AMF‐Triggered Release

**DOI:** 10.1002/smll.74972

**Published:** 2026-08-10

**Authors:** Ana Maria Panaite, Aleksandra Predeina, Aitor Alvarez Lorenzo, Manuel Ceballos, Pascal Clerc, Niccolò Silvestri, Andrea Griesi, Giorgio Divitini, Julian Carrey, Veronique Gigoux, Pablo del Pino, Teresa Pellegrino

**Affiliations:** ^1^ Istituto Italiano di Tecnologia (IIT) Genoa Italy; ^2^ Universita degli Studi di Genova (UniGe) Genoa Italy; ^3^ Departamento De Física de Las Partículas, Centro Singular de Investigación en Química Biolóxica e Materiales Moleculares (CiQUS) Universidade De Santiago de Compostela Santiago de Compostela Spain; ^4^ CRCT CNRS Centre de Recherches en Cancérologie de Toulouse Université Toulouse III‐Paul Sabatier Université De Toulouse Toulouse France; ^5^ Laboratoire De Physique et Chimie des Nano‐Objets (LPCNO), INSA, CNRS Toulouse France

**Keywords:** composites, controlled drug release, magnetic hyperthermia, magnetic nanoparticles, metal‐organic framework (MOF)

## Abstract

A seeded growth strategy was developed to synthesize core–shell magnetic metal–organic framework (MOF) composites for magnetic hyperthermia (MHT) and MHT‐triggered drug delivery. Cubic or spherical iron oxide nanoparticles, with nanocubes selected for their superior MHT performance, were coated with cetyltrimethylammonium bromide to enable aqueous ZIF‐8 shell growth. Shell thickness strongly influenced heating efficiency under alternating magnetic fields (AMFs), with thinner shells and cubic cores yielding enhanced MHT performance. Doxorubicin (Doxo) was used as a model chemotherapeutic drug and loaded either by surface adsorption or via in‐situ encapsulation during ZIF‐8 growth, the latter achieving an exceptional loading efficiency of 98%. To ensure stability in physiological environments, an amphiphilic polymer coating was applied, improving dispersion while regulating shell degradation and drug release. Doxo‐loaded composites exhibited efficient cellular uptake and lysosomal localization in glioblastoma and breast cancer cells. Confocal microscopy revealed that magnetic field exposure induced lysosomal permeabilization and redistribution of Doxo, indicating a potential lysosomal escape mechanism. Notably, enhanced cytotoxicity occurred only when AMFs were applied to Doxo‐loaded composites, despite no measurable bulk temperature increase, suggesting localized MHT‐induced intracellular damage. Overall, shell‐tunable magnetic‐MOF nanohybrids emerge as promising platforms for controlled, heat‐free intracellular drug activation for targeted cancer therapy.

## Graphical abstract

We developed core‐shell iron oxide@ZIF‐8 magnetic‐MOF nanohybrids via a seeded growth method for magnetic hyperthermia‐triggered drug delivery. Cubic cores and thinner shells maximized heating. In‐situ doxorubicin encapsulation achieved 98% loading, better than surface encapsulation. Polymer coating improved stability. An in‐vitro cell study reveals that alternating magnetic fields prompted lysosomal permeation, with drug redistribution thus enhancing cancer cell killing without measurable bulk heating.

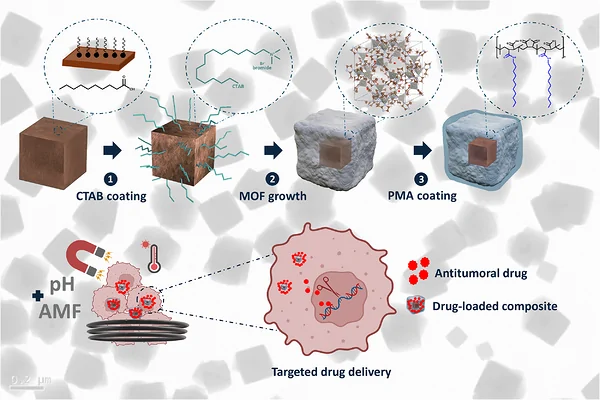

## 1 Introduction

In the race against cancer, chemotherapy remains the first‐line therapy. However, it suffers from several limitations, including insufficient drug delivery to the tumor site, difficulties in penetrating cell membranes, off‐target toxicity, and problems with drug solubility and degradation, leading to undesirable side effects and drug resistance [1, 2, 3]. To address these limitations, there is an increased focus on developing biocompatible, degradable, and smart nano‐vectors that can load and deliver chemotherapeutic drugs, with more precision, to the tumor site [4, 5, 6].

Stimuli‐responsive drug delivery systems have emerged as a powerful tool to overcome limitations of standard chemotherapy since they provide spatiotemporal release control over conventional therapeutics. In these systems, drug release is activated only in response to a specific stimulus, enabling high local drug concentrations at the tumor site while minimizing systemic toxicity. These stimuli fall into two categories: endogenous and exogenous [5]. Endogenous stimuli exploit physiological differences between tumor environment and healthy tissues, such as the acidic pH at the tumor site, reducing tumor environment, such as glutathione, or abundant presence of enzymes, such as lytic enzymes. Conversely, exogenous stimuli are controlled remotely, by external stimuli applied outside the body, such as light, temperature, ultrasound, or magnetic fields. The combination of endogenous and exogenous stimuli can enable precise temporal control (release only when the stimulus is applied) and spatially selective (stimulus applied only to the targeted area), offering the possibility of a personalized on‐demand drug release [7, 8, 9]. Among these, magnetic stimuli based on kHz radiofrequencies are particularly attractive due to their ability to penetrate deep into the body without signal attenuation and under patient‐safe magnetic field conditions.

Magnetic nanoparticles (MNPs) such as iron oxide (Fe_3_O_4_) nanoparticles (NPs) are well‐established efficient magnetothermal agents when exposed to an alternating high‐frequency magnetic field (AMF) [10, 11, 12, 13]. Heat generation arises from a combination of Neel and Brownian relaxation processes, and the relative contribution of each mechanism is governed by nanoparticle features, including size, magnetic anisotropy, interparticle interactions, and external parameters, such as viscosity of the tumor microenvironment or the presence of a shell material around the magnetic core [14, 15]. These factors collectively determine the specific absorption rate (SAR) value, which measures the key magneto‐heat conversion rate for magnetic hyperthermia (MHT) performance [16, 17]. SAR efficiency can be optimized by tailoring size, shape, surface coating, and composition of Fe_3_O_4_ NPs to maximize magnetic losses within clinically safe AMF parameter conditions (requiring that the product of the field intensity (H) and frequency (*f*) remain below 5 × 10^9^ Am^−1^s^−1^) [14, 18, 19].

On the other hand, metal‐organic frameworks (MOFs), which are a class of highly porous materials assembled by metal ions or metallic clusters known as secondary building blocks (SBU) with organic linkers, exhibit excellent drug interactions and high loading thanks to the ability to tune their structures and porosity [20, 21]. Since their first use in 2006 [22], MOFs have been extensively proposed for drug delivery applications [23, 24] and, more recently, stimuli‐responsive MOFs have gained major attention, as they have the ability to trigger drug delivery when subject either to internal cellular factors like adenosine triphosphate (ATP), redox reactions, and pH, or to extracellular stimuli such as temperature, pressure, irradiation, and magnetic fields [25, 26, 27, 28]. Specifically, ZIF‐8 is a MOF known for its high drug‐loading capacity, pH stimulus response, and relatively high biocompatibility of the MOF units, the Zn and imidazole linkers [29]. Given the acidic environment of the tumor microenvironment with a pH around 6.5, and sub‐compartments of tumor cells with endosomes having a pH of 5.5 – 6.0 and lysosomes having a pH of 4.5 – 5.0, the pH response of the ZIF‐8 MOF is provided by the imidazole protonation at acidic pH, resulting in disassembly of the ZIF‐8. Several payloads, including Doxorubicin (Doxo) [30, 31], 5‐fluorouracil (5‐FU) [32], caffeine [33], Deoxyribonucleic acid (DNA) [34], to cite a few examples, have been successfully encapsulated using either the in‐situ encapsulation during the synthesis or the post‐synthesis encapsulation methods [35]. Moreover, its synthesis is straightforward and can be performed in aqueous media under mild conditions, enhancing its suitability for scalable and biocompatible drug delivery formulations [36].

The strategic combination of MNPs with MOFs thus enables the development of drug delivery systems (DDSs) that can release their cargo in a controlled manner via conformational changes or be triggered upon application of AMF. Only a few works have proposed the development of composites based on Fe_3_O_4_ NPs and ZIF‐8 for encapsulation and controlled release of drugs, while most research to date is focused on plasmonic NPs [37, 38]. For example, Yang et al.  [39] proposed this same approach for the formation of ZIF‐8 MOF on the surface of polydopamine (PDA) coated cobalt ferrite (CoFe_2_O_4_) NPs, resulting in a CoFe_2_O_4_@PDA@ZIF‐8 composite. The mesoporous cobalt ferrite NPs pre‐synthesized by a solvothermal method were first coated with dopamine under an alkaline environment (in tris buffer at pH 8.5), promoting PDA polymerization. The PDA not only gives stability to the NPs, avoiding their aggregation, but also facilitates the heterogeneous growth of ZIF‐8 by coordination via catechol groups present in PDA with Zn^2+^ ions to provide a template on which ZIF‐8 could nucleate and grow in crystal structures in a short 30 min reaction at room temperature (RT). The resulting 150 nm flower‐like composite showed a superparamagnetic behavior at RT with a saturation magnetization (M_s_) of 36 emu g^−1^, smaller than the 68.5 emu g^−1^ of the pristine CoFe_2_O_4_. In another work, Ruixin Bian et al.  [40] successfully achieved the multi‐step assembly of a multilayered structure, starting with an iron oxide core and by using an intermediate polymer, which formed beads on the Fe_3_O_4_, to then introduce Au NPs on the polymer beads and promote the growth of ZIF‐8 on the gold surface. As a last step, the post‐encapsulation of Doxo on the preformed assembly enabled it to reach a high loading efficiency of 1.54 mg Doxo per mg of composite. The system was studied as drug carrier for tumor chemotherapy and as bi‐modal imaging system, exploiting the Fe_3_O_4_ based‐signal for MRI, and the Au‐based signal for computed X‐ray tomography [40]. Chen et al.  [41] reported a simplified core‐shell Fe_3_O_4_@ZIF‐8 system and used a comparable post‐synthesis Doxo encapsulation approach to achieve a loading efficiency of 77% while reaching a high Doxo loading content of 0.33 mg per 1 mg of ZIF‐8. The resulting 180 nm flower‐like composite showed a superparamagnetic behavior at RT with an M_s_ of 37 emu g^−1^, smaller than the 48 emu g^−1^ of the pristine Fe_3_O_4_. No further coating shell was used, and the release was studied at pH 5.5 and 7.4, showing a boosted release of Doxo of 63% after 48 h. Such rapid release may limit the capacity to achieve prolonged or stimulus‐selective delivery. On the other hand, the toxicity of the composite was assessed by MTT assay using Hepatoma cells, showing no toxicity up to 100 µg mL^−1^.

Despite these promising examples, significant gaps remain in the development of Fe_3_O_4_@ZIF‐8 composites for AMF‐triggered drug delivery. The influence of NPs size and shape on correlated magnetic performance, on magnetic heat losses, and drug delivery efficiency depending on the chosen loading methods has not been addressed. While well‐defined core‐shell structures have been successfully achieved with plasmonic and catalytic NPs [42, 43, 44], obtaining uniform ZIF‐8 coating on magnetic cores remains challenging, especially without compromising their intrinsic magnetic properties. Furthermore, magnetic characterization of such systems in literature is often limited to include Superconducting Quantum Interface Device (SQUID) measurements, generally restricted to RT measurements, overlooking valuable information such as low‐temperature magnetic behavior and superparamagnetic blocking temperatures, often lacking quantification of magnetic heat losses at high frequency. Furthermore, there is a need for streamlined synthesis protocols that balance ease of production with preservation of magnetic properties, as well as for systematic studies correlating the structural parameters of the composites, such as shell thickness and crystallinity, with magnetic hyperthermia efficiency and controlled drug release kinetics [45]. Another critical limitation of ZIF‐8 is its limited chemical and structural stability in aqueous environments due to the presence of CO_2_ dissolved in water, which decomposes the framework, forming ZnCO_3_ [46], hindering its effectiveness as DDSs. To address this issue, further functionalization of these composites with amphiphilic polymers is necessary to inhibit degradation and enhance stability in aqueous media. Addressing these aspects is essential to fully elucidate the structure‐property relationship that governs AMF‐triggered release performance.

In this work, we propose a magnetic Fe_3_O_4_@ZIF‐8 core‐shell (CS) composite as carrier for potential tumor therapy with chemotherapy effects enhanced upon exposure to AMF. To our knowledge, the use of such composites for combining chemotherapy with exposure to AMF without any macroscopic temperature increase as a controlled release mechanism has not been explored yet. This work also fills the gap in the current synthesis and scale‐up approach of Fe_3_O_4_@ZIF‐8 composites. The study aims to compare spherical and cubic shapes of pre‐synthesized Fe_3_O_4_, the latter with benchmark MHT heating properties, both coated with MOF by a two‐step seeded growth approach comprising the water transfer of MNPs with cetyltrimethylammonium bromide (CTAB) and the further nucleation of ZIF‐8 on their surface in aqueous conditions with tunable ZIF‐8 thickness. Comprehensive structural and magnetic characterizations reveal not only a well‐controlled core‐shell structure but also their peculiar viscosity‐independent magnetic heat losses across composites at different shell thicknesses. Next, the coating with poly (isobutylene‐alt‐maleic anhydride) graft‐dodecyl (PMA) was needed to better stabilize them in aqueous media and control the kinetics of release of the Doxo. For Doxo encapsulation, two approaches, including an in‐situ protocol of drug encapsulation during the ZIF‐8 growth and the more standard post‐synthesis covering, were compared, revealing a higher encapsulation efficiency with the new in‐situ method. On these Doxo‐loaded composites, stability in water, physiological conditions, and complete Dulbecco's Modified Eagle Medium (DMEM) medium suggest a slower structural degradation, hence, a slower drug release process. Finally, the AMF exposure as a mechanism to induce the release of Doxo on tumor cells was investigated either by in‐situ confocal study on single cells under AMF exposure or indirectly by comparative viability studies on U‐87 glioblastoma cells and MDA‐MB‐468 breast cancer cells, revealing the enhancement of cytotoxic effects always in the presence of a time‐varying field.

## 2 Results and Discussion

### 2.1 Core‐shell Fe_3_O_4_@ZIF‐8 Composites Synthesis

The main nanoparticles selected as core for the formation of the Fe_3_O_4_@ZIF‐8 composites (CS) are cube‐like iron oxide nanoparticles (IONPs) (Figure 1), owing to their outstanding magnetic properties [47]. These selected nanoparticles (NPs) were synthesized via a thermal decomposition method previously reported by our group [12, 13], rendering highly monodispersed samples of cube edge of 18 ± 1 nm and coated by decanoic acid as organic surfactant for their stabilization in chloroform (Figure 1A,B, and Figure S1A,E).

**FIGURE 1:**
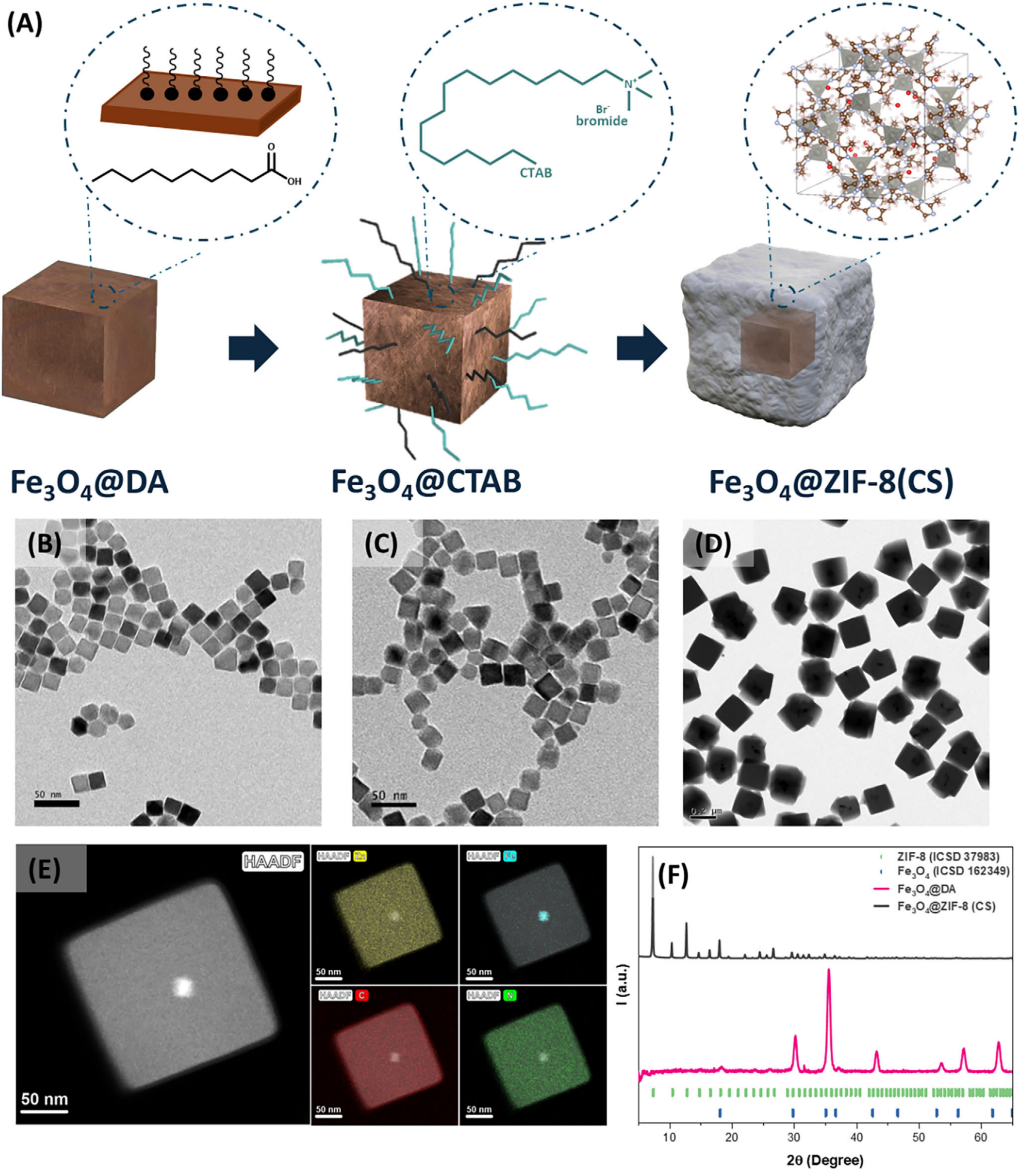
(A) Schematic illustration of the synthesis of the Fe_3_O_4_@ZIF‐8 core‐shell composites (CS). TEM images of each step of the reaction. (B) TEM image of cubic Fe_3_O_4_@DA; (C) cubic Fe_3_O_4_@CTAB; and (D) CS; (E) High‐angle annular dark‐field STEM (HAADF‐STEM) image of CS and corresponding STEM‐EDS elemental maps for single element Zn (yellow), Fe (turquoise), C (red) and N (green); and (F) PXRD pattern on the CS in MeOH and Fe_3_O_4_@DA compared to Fe_3_O_4_ (ICSD 162349) and ZIF‐8 (ICSD 37983) patterns.

The synthesis of the required composites was carried out using a simple procedure in water, in the presence of Zn(NO_3_)_2_, 2‐methylimidazole (MeIM) as organic linker, and with NPs coated with hexadecyltrimethylammonium bromide (CTAB) as a capping agent, as outlined in previous studies (Figure 1A) [37, 38]. CTAB, besides providing stability in water, which is the solvent in which the ZIF‐8 will grow, also acts as both size‐controlling agent and structural directing agent of ZIF‐8 crystals [44].

This synthetic route, when applied to IONPs, has proven to be sensitive to some parameters, particularly the CTAB amount used during the coating process of the NPs. To optimize the optimal amount of CTAB per NP and to ensure the formation of well‐controlled composites with size <200 nm, considered ideal for the extravasation of the composite to tumors, the amount of CTAB molecules/nm^2^ was tuned: at fixed cubic NPs concentration of 0.18 µM, 1 mL of CTAB at different concentrations 0.05 M, 0.1 M, and 0.2 M, corresponding to 1000, 1866, and 3731 molecules/nm^2^, respectively. The ratio of 1000 molecules/nm^2^ yielded the most favorable results, minimizing the formation of micelles, as clearly visible in Figure S2A. Transmission Electron Microscopy (TEM) of the nanocubes revealed a very similar shape to that of decanoic‐coated nanocubes with a different shadow at the edge, likely due to the thicker layer of CTAB on top of decanoic acid (Figure 1C). In these experiments, the CTAB/NPs ratio was kept constant at 1000 molecules/nm^2^, ensuring reproducibility and preventing excess surfactant aggregation. The dynamic light scattering (DLS) graphs in Figure S1I show that the NPs have a monomodal hydrodynamic size distribution and high stability, as indicated by the low polydispersity index (PdI) (0.285) and the hydrodynamic sizes by number‐weighted (d_h,N_) of 41 ± 4 nm, confirming the absence of aggregates (Table S1). TEM analysis further corroborates these results, showing monodispersed magnetic cores of 18 ± 1 nm.

In order to further confirm the successful coating process, comparative FT‐IR characterization was performed on the nanoparticles and the CTAB ligand used in the surface functionalization, as shown in Figure S1L. FT‐IR spectra show signals for O─H stretching (3500 cm^−1^, see purple colored region), C═O stretching (1700 cm^−1^, see green colored region), corresponding to the absorbed water on the structure [48] The good match between the signals C─H stretching (2965 and 2878 cm^−1^, see orange colored region) attributed to ─CH_2_ groups present in CTAB, and the C─N stretching vibrations at 1100 cm^−1^ (see yellow colored region) confirms the successful coating with CTAB. To note that in this CTAB exchange step, as well as on the next one of ZIF‐8 growth, an important precaution to take into consideration was to avoid the use of magnetic stirring, due to the aggregation of magnetic materials on top of the stirrer bars (Figure S2). For this reason, an ultrasonic bath was used to energetically mix the MNPs with the CTAB at 60°C, minimizing aggregation (Figure S2C). Following this procedure, the NPs exhibited improved colloidal stability after washing, as confirmed by DLS, showing a decrease in the hydrodynamic sizes by intensity‐weighted (d_h,I_) from 168 ± 16 nm to 66 ± 4 nm and PdI from 0.323 to 0.180 (Figure S2B,D).

As a second step, the CTAB‐coated IONPs were used as seeds for the ZIF‐8 shell crystallites growth. The influence of the order in which the metal and the ligand were added was investigated. The successful synthesis of the composites occurs by mixing Zn^2+^ with MeIM and NPs coated with CTAB, as previously reported for AuBy@ZIF‐8 [38]. The order in which the reactants were added was of crucial importance: if the MeIM ligand was added first, followed by the addition of Zinc ions and then the CTAB‐coated NPs in aqueous media, the core‐shell composite grows in a monodispersed cubic shape (Figure S3B). In contrast, if the Zn (II) was added first, then the ligand and finally the NPs solution, the composites had an irregular shape (Figure S3A). The resulting core‐shell composite also exhibits a lower d_h,I_ of 313 ± 16 nm versus 670 ± 12 nm obtained following the first route of synthesis (Figure S3C and Table S2).

Therefore, for the optimized protocol, the reaction mixture was sonicated in a water bath in order to ensure a homogeneous solution. Then, the mixture was left undisturbed for 3 h at RT to promote the growth of the MOF. The obtained composite was concentrated and washed by the addition of MeOH and centrifugation (7000 rcf for 10 min) with final redispersion in MeOH (1 mL) to preserve the MOF structure.

TEM images showed that the MNPs were at the center of cubic‐like ZIF‐8 polycrystals, resulting in a homogenous core‐shell heterostructure of 232 ± 6 nm (Figure 1D) as expected in the case of the formation of a polycrystalline ZIF‐8 shell, facilitated by the presence of CTAB molecules on the IONPs surface. This structural arrangement, also evident from the tomography video (Video S1), which clearly reveals the Fe_3_O_4_ core embedded within the MOF shell, is consistent with the expected formation of the polycrystalline ZIF‐8 shell, facilitated by the presence of CTAB molecules on the IONPs surface.

The formation of the composite was further analyzed by scanning TEM energy dispersive X‐ray spectroscopy (STEM‐EDS) (Figure 1E and Figure S4). The compositional maps of C, O, Fe, and Zn elements fully support the core‐shell arrangement with the Fe_3_O_4_ nanocubes as a core and a complete ZIF‐8 shell. Furthermore, the average concentration in the composites measured by EDS matched very well the composition measured via elemental analysis using ICP‐OES (Table S3).

The crystalline structure of all the composites was also confirmed by Powder X‐ray diffraction (PXRD) analysis (Figure 1F). The obtained diffraction pattern indicated the presence of pure‐phase ZIF‐8. The relative intensity of diffraction peaks is indexed as ZIF‐8 (ICSD 37983). Furthermore, the sharp and well‐defined peaks observed in the PXRD pattern suggest that the CS is highly crystalline. The absence of any additional peaks indicated that there are no impurities or secondary phases present in the samples. The characteristic peaks were observed at 10.4°, 12.7°, 14.7°, 16.5°, 18°, and 19.5°, corresponding to (002), (112), (022), (013), (222), and (123), respectively. However, the diffraction peaks of magnetite powder (ICSD 162349) are not visible without a detailed analysis of the diffractogram, due to the overlap between the main diffraction peaks of Fe_3_O_4_ and the multiple reflections of ZIF‐8. This effect is further accentuated by the higher proportion of ZIF‐8 in the shell, which reduces the relative intensity of the Fe_3_O_4_ reflections compared to those of the MOF. Pawley refinement analysis revealed overlapping and very broad diffraction peaks (Figure S5, **zoom**) corresponding to Fe_3_O_4_, consistent with the small crystalline domains of the IONPs resulting from their nanoscale size.

### 2.2 Scale‐Up Protocol of Core‐Shell Fe_3_O_4_@ZIF‐8 Composites

Scaling up the synthesis of colloidal nanoparticles and streamlining their post‐synthetic processing are essential for reducing costs and time, thereby facilitating their utilization for characterization and use for in‐vitro and preclinical trials. To this aim, we set a procedure using a robotic liquid handler (Nimbus Hamilton) [49]. The Nimbus system offers the advantage of performing multiple reactions simultaneously, thus saving time and ensuring reproducibility. To implement the scaling up, a straightforward replication of the ZIF‐8‐bench step was performed, with the only exception that, with Nimbus, an orbital shaker was employed as a method to mix the reaction components instead of using the sonication step (Figure 2A). First, three stock solutions were prepared containing MeIM (1.3 M), Zn(NO_3_)_2_ (0.025 M), and Fe_3_O_4_@CTAB (2 nM). Then, the automated procedure began by dispersing 1 mL of MeIM into each of the 24 glass vials (8 mL volume capacity) under an orbital shaker at 500 rpm, which was maintained throughout the process. This was followed by the addition of 1 mL of zinc nitrate solution (0.025 M) and finally 1 mL of the CTAB‐capped NPs (2 nM in NPs and at 0.2 mg mL^−1^ of CTAB). After completing the addition, the solutions were kept under shaking for 5 min and then left undisturbed at RT for 3 h. Indeed, it can be observed that the CS are monodispersed and fully dispersed in MeOH as they have a similar monomial size distribution with respect to the sample synthesized on‐bench (Figure 2B). Their d_h,N_ are very similar too, 225 ± 8 nm versus 237 ± 7 nm, confirming the reproducibility of the automated process and indicating that this scaling up protocol can be successfully used for the formation of the CS (Figure 2C). In contrast, we did not apply the automated procedure for the CTAB‐coating prior to the growth of ZIF‐8, since our strategy proved highly effective, as the NPs remained colloidally stable in suspension for up to 1 year without visible aggregation or sedimentation.

**FIGURE 2:**
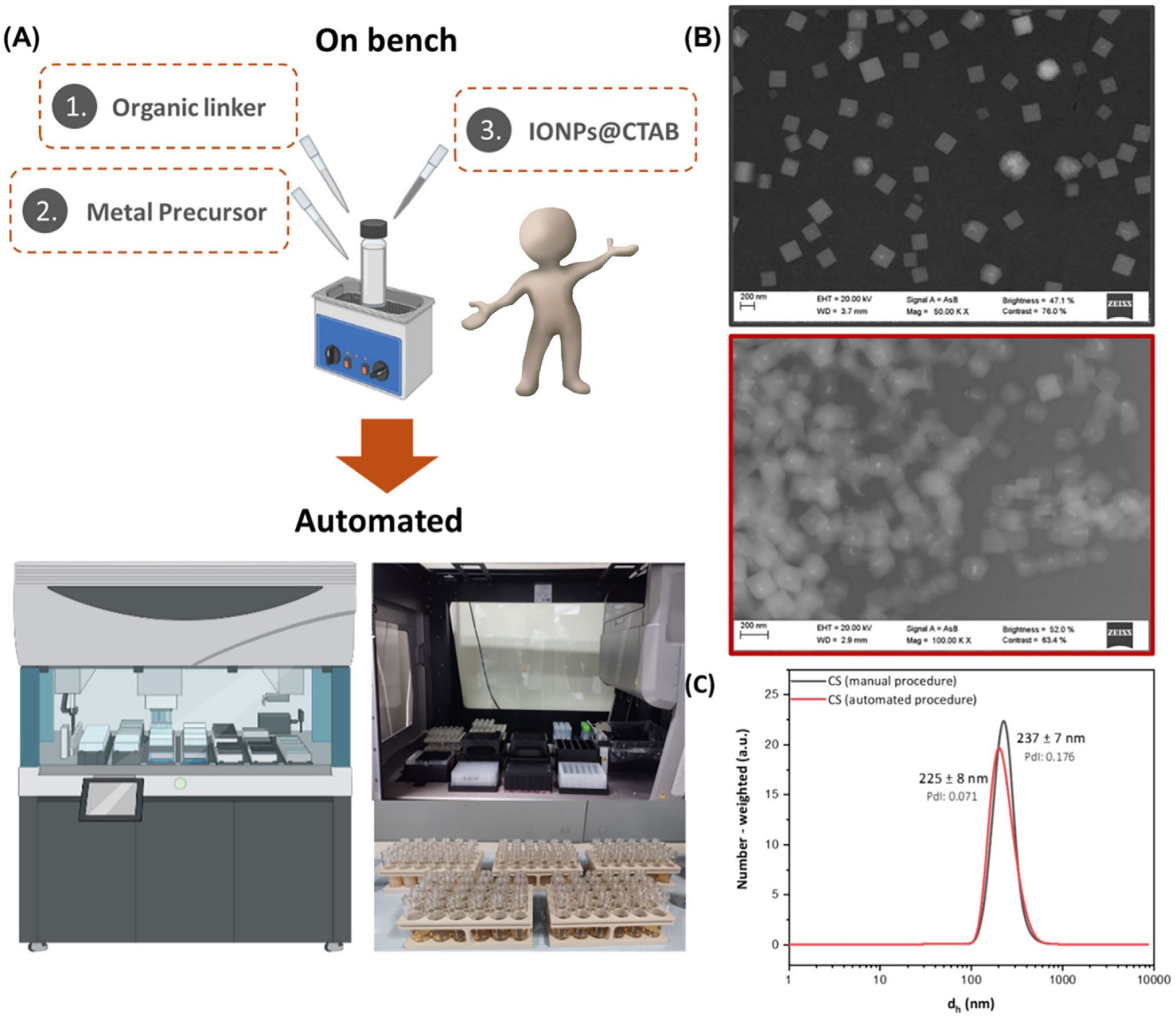
(A) Schematic representation of the On‐bench (up) vs the Automated procedure (down) to produce CS composites with a photograph showing the Nimbus platform and arm, and a photograph of 5 batches of nanoparticles synthesized with Nimbus; (B) SEM images of CS composites made by manual procedure (black frame) and made by automated procedure (red frame); and (C) DLS graphs showing the hydrodynamic sizes by number‐weighted (d_h,N_).Hydrodynamic diameters (d_h_) (mean value ± standard deviation SD). Image was made with Biorender.

Through these automated procedures, we were able to produce 24 batches of composites per run, maintaining the order of addition of the bench protocol and, in comparison to the bench protocol, which typically allows for the preparation of 10 samples in 1 h, the automated routine enables us to fabricate 120 samples in less than 90 min.

### 2.3 Implementation of the Core‐Shell Fe_3_O_4_@ZIF‐8 Composite on Another Magnetic Core

Aiming to expand the portfolio of CS having magnetic cores of different shapes and study their comparative MHT performances, we implemented the CS synthesis on spherical iron oxide NPs having the same volume as the cubic NPs. This enables a direct evaluation of the effects of the shape on their magnetic properties for a fixed volume. The selected nanoparticles were synthesized via a thermal decomposition method already reported in our group [50], using oleic acid to stabilize the NPs, rendering highly monodispersed samples of 22 ± 2 nm (Figure S1B,F). As with cube‐like NPs, the spherical NPs were coated with CTAB, keeping the same optimized conditions used for the cubic cores. TEM confirmed that the CTAB coating did not alter the size or morphology of the NPs (Figure S1D,H). Furthermore, the d_h,N_ was measured to be 55 ± 5 nm, indicating good colloidal stability, comparable with the cubic ones (Figure S1J).

The colloids were stable in solution for approximately one year, thanks to the steric stabilization provided by CTAB molecules. The ζ‐potential values were in the range of +45 ± 2 mV for cubic and + 32 ± 2 mV for spherical Fe_3_O_4_ (Figure S1K). Notably, the cubic‐shaped Fe_3_O_4_ exhibits higher values of SAR (80 ± 1 W/gFe) with respect to the spherical‐shaped NPs (19 ± 1 W/gFe), due to the inherent shape anisotropy, and they exhibit high SAR values even at clinical frequencies (110 kHz, 16 KA m^−1^) (Figure S6). As expected, the SAR values increased with both frequency and field. A summary of the SAR values measured at different fields and frequencies is provided in Table S4.

### 2.4 Shell Effect on Magnetic Heat Losses for Core‐Shell Fe_3_O_4_@ZIF‐8 Composites

Aiming at systematically investigating the influence of varying MOF shell thickness on the properties of our magnetic NPs, the influence of the CTAB concentrations as thickness regulating agent was also studied for both kinds of magnetic NPs (Figure 3). For this, the NPs were dispersed in 1 mL CTAB solutions of 0.2, 0.4, and 0.6 mg mL^−1^, to obtain a final concentration of the NPs of 4 nM. A reduction in size is observed from SEM images (Figure 3) from 188 ± 18 to 123 ± 9 nm in the case of using cubic Fe_3_O_4_ as core, and from 170 ± 15 to 132 ± 9 nm in the case of using spherical Fe_3_O_4_ as core (Figure S7). This reduction in size is also measurable from the DLS graphs (Figure S8A,D). The d_h,N_ decreases from 232 ± 6 nm to 200 ± 9 nm in the case of using cubic Fe_3_O_4_ as core, and from 246 ± 6 nm to 197 ± 9 nm in the case of using spherical Fe_3_O_4_ as core. This is due to the increased number of CTAB molecules absorbed onto the ZIF‐8 crystal surface, which are slowing down the growth rate of the MOF.

**FIGURE 3:**
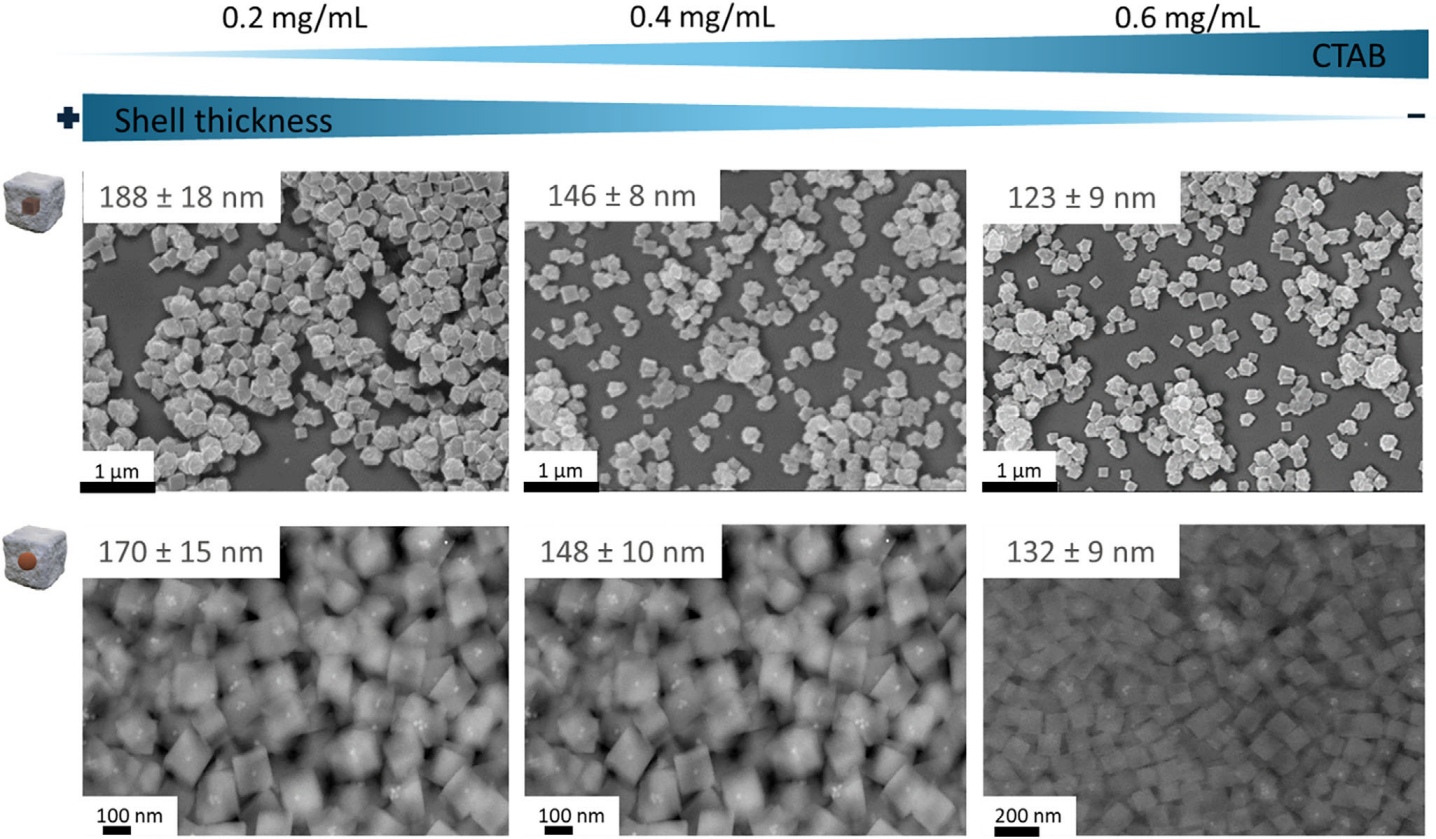
Influence of the increase of the concentration of CTAB in the MOF shell; SEM images showing the decrease in size of the composite using cubic Fe_3_O_4_ core (upper region); and spherical Fe_3_O_4_ core (lower region).

The crystalline structure of all composites was confirmed by PXRD, which consistently showed sharp, well‐defined peaks corresponding to pure phase ZIF‐8 (ICSD 37983), indicating high crystallinity and the absence of impurities or secondary phases. As expected, diffraction peaks from the magnetite cores (ICSD 162349) were not visible, without a detailed analysis of the diffractogram, due to the overlap between the main diffraction peaks of Fe_3_O_4_ and the multiple reflections of ZIF‐8. This effect is further accentuated by the higher proportion of ZIF‐8 in the shell, which reduces the relative intensity of the Fe_3_O_4_ reflections compared to those of the MOF, as previously observed (Figure S8B,E).

Raman spectroscopy was employed to analyze the different composites as well as pristine ZIF‐8, with CTAB‐coated Fe_3_O_4_ NPs (both cubic and spherical) serving as controls. The Raman spectra revealed that the magnetic composites exhibited prominent characteristic vibration modes of ZIF‐8, including imidazole ring puckering (685 cm^−1^), C–N stretching (1146 cm^−1^), and C–H wagging (1460 cm^−1^) (Figure S8C,F, blue‐highlighted regions). In contrast, the bands corresponding to Fe_3_O_4_ NPs were less discernible, which can be attributed to the dominant ZIF‐8 signals. However, some features associated with the Fe_3_O_4_ were observed at approximately 670, 300, and 500 cm^−1^, corresponding to the A_2g_ and 2T_2g_ modes of magnetite crystalline structure, respectively (Figure S8C,F, purple‐highlighted regions).

Thermogravimetric analysis (TGA) under air of the pristine ZIF‐8 and the prepared composites with both cubic and spherical cores showed a sharp weight loss (68 wt.%) at around 400°C, associated with the total combustion of MeIM and the formation of different inorganic residues of ZnO (Figure S9A,C). However, in the case of composites, weight loss occurred at slightly higher temperatures, likely due to the presence of the oleic acid/CTAB or decanoic acid/CTAB residues on the Fe_3_O_4_ surface. The ZIF‐8 acts as a thermal insulator that delays degradation, pushing it to higher temperatures [37]. The different inorganic residues enable us to estimate the relative content of Fe_3_O_4_ in the samples, as shown in Table S5.

The gas adsorption capacities of the pristine ZIF‐8 and the composites were assessed through N_2_ adsorption isotherms at 77 K, all of which exhibited typical type I isotherms characteristic of microporous materials (Figure S9B,D) [51]. Pristine ZIF‐8 showed an N_2_ uptake of 410 cm^3^/g corresponding to a Braunauer‐Emmet‐Teller (BET) surface area of 1601 m^2^/g. The corrected S_BET_ values of the composites obtained by subtracting the Fe_3_O_4_ mass fraction determined from TGA analysis (Table 1), fall within a similar range, confirming that the porosity of the ZIF‐8 shells is preserved after composite formation (Figures S10–S16).

**TABLE 1:** S_BET_ values for ZIF‐8, CS‐S1 (spherical, 170 nm), CS‐S2 (spherical, 148 nm), CS‐S3 (spherical, 132 nm), CS‐C1 (cubic, 188 nm), CS‐C2 (cubic, 146 nm), and CS‐C3 (cubic, 123 nm).

Sample	BET surface area (m^2^/g)	Corrected BET surface area (m^2^/g)^*^	wt % Fe_3_O_4_
ZIF‐8	1601	1601	n/a
CS‐S1 (spheres, 170 nm)	1530	1570	2.57
CS‐S2 (spheres, 148 nm)	1321	1359	2.83
CS‐S3 (spheres, 132 nm)	1058	1061	0.29
CS‐C1 (cubes, 188 nm)	1186	1221	2.83
CS‐C2 (cubes, 146 nm)	1114	1132	1.57
CS‐C3 (cubes, 123 nm)	1252	1294	3.26

* Corrected Considering % Fe_3_O_4_

Static magnetic properties were obtained by recording the hysteresis loops with finite coercive fields (H_c_) and temperature‐dependent magnetization for the composites (Figure 4).

**FIGURE 4:**
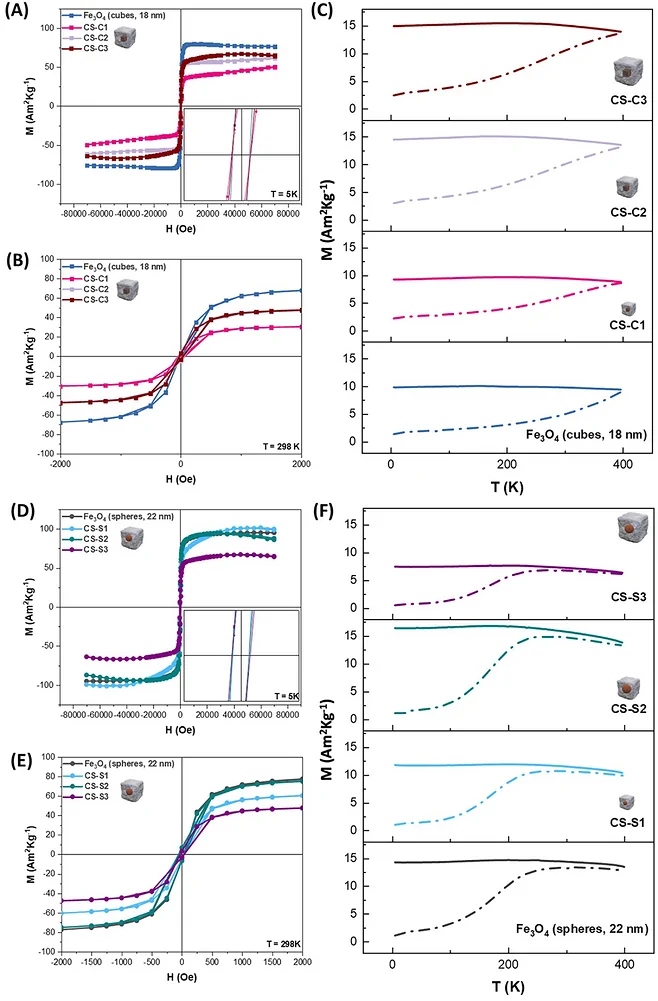
Magnetization vs applied magnetic field curves normalized to the Fe amount of cubic Fe_3_O_4_ and its composites at 5 K (A) and 298 K (B); and of spherical Fe_3_O_4_ and its composites at 5 K (D) and 298 K (E), respectively. Zero‐Field cooled and Field cooled (ZFC‐FC) of Fe_3_O_4_ (C) cubic and its composites, and (F) spherical and its composites.

Normalization of the saturation of magnetization (M_s_) was made considering the Fe contribution and eliminating the MOF contribution. It can be observed that the M_s_ at 5 K of the CS increases with respect to the spherical Fe_3_O_4_ core (Figure 4A–D), from 54 Am^2^/kgFe (Fe_3_O_4_ spheres) to 64–100 Am^2^/kgFe (CS), reaching the highest value, 100 Am^2^/kgFe when the ZIF‐8 shell is smaller (CS‐S3, 170 nm, when using 0.6 mg/mL CTAB) (Table 2). Conversely, for the cubic Fe_3_O_4_ core, the M_s_ decreases from 95 Am^2^/kgFe (Fe_3_O_4_ cubic) to 34–59 Am^2^/kgFe (CS) with the lowest value observed when bigger is the shell (CS‐C1, 188 nm, when using 0.2 mg/mL CTAB) (Figure 4A–D). This effect is due to the surface contribution to the surface anisotropy (K_s_), originating from the spin direction discrepancy between core and surface [52]. The arrangement of the magnetic moments within the core‐shell can be influenced by the interface between the core and the shell, similarly to what happens in Fe_3_O_4_‐SiO_2_ structures [53]. This misalignment is more pronounced in the case of the cubic core, because cubes have higher surface‐to‐volume ratio than the spheres, resulting in more atoms exposed on the surface for interaction with the ZIF‐8 shell. This increased exposure amplifies surface anisotropy effects. Moreover, the surface atoms may have a different coordination environment, resulting in stronger interaction with ZIF‐8 Shell. This can be corroborated with the low M_s_ at 298 K (31 Am^2^/kgFe) and 5 K (34 Am^2^/kgFe) obtained when bigger is the shell of ZIF‐8 (CS‐C1, 188 nm, 0.2 mg/mL CTAB).

**TABLE 2:** Magnetic parameters determined by static measurements obtained through recording hysteresis cycles at 5 and 298 K of the Fe_3_O_4_ (cubic), Fe_3_O_4_ (spherical), and their corresponding CS composites.

Sample	298 K		5 K		T_max_ (K)
	M_s_ (Am^2^/kgFe)	H_c_ (Oe)	M_s_ (Am^2^/kgFe)	H_c_ (Oe)	
Fe_3_O_4_ (spherical, 22 nm)	62	8	54	275	296
CS‐S1 (spheres, 170 nm)	76	30	100	266	266
CS‐S2 (spheres, 148 nm)	60	35	86	272	266
CS‐S3 (spheres, 132 nm)	48	40	64	310	266
Fe_3_O_4_ (cubic, 18 nm)	103	25	95	260	396
CS‐C1 (cubes, 188 nm)	31	69	34	328	397
CS‐C2 (cubes, 146 nm)	47	57	55	306	397
CS‐C3 (cubes, 123 nm)	47	39	59	300	396

At RT (Figure 4B,E), both the NPs and the composites showed superparamagnetic behavior, as observed from the negligible coercivity (H_c_) below 100 Oe. Regarding the zero‐field cooled (ZFC)—field cooled (FC) (Figure 4C,F), we can observe that the mean blocking temperature (T_max_) of sphere and cubic‐shaped NPs is very different, being that of cubic‐shaped NPs blocked at higher temperatures (Table 2).

To better understand the behavior of these composites, normalization of the M_s_ was also attempted based on Fe+Zn amount (Figure S17). It was observed that the M_s_ at 5K for the composites drastically decreased when spherical Fe_3_O_4_ were used, dropping from 54 Am^2^/kgFe (Fe_3_O_4_ sphere) to 8 Am^2^/kgFe when embedded in a 170 nm ZIF‐8 shell (CS‐S1, 170 nm). A similar trend was observed for CS with cubic Fe_3_O_4_ core, where M_s_ decreased from 95 Am^2^/kgFe to 0.6 Am^2^/kgFe (CS‐C1, 188 nm). This substantial reduction in M_s_ is mainly attributed to the high paramagnetic contribution of zinc, given the fact that the mass of the two components is drastically different: 1.021 mg Zn vs 0.05 mg Fe.

### 2.5 Colloidal Stability in Aqueous Media

Although previous studies have reported core‐shell metal nanoparticles encapsulated in ZIF‐8 using different surfactants or diverse nanoparticle cores, it is crucial to acknowledge the well‐documented issue of ZIF‐8's instability in aqueous environments [37, 38]. In this respect, our MOF composites exhibited long‐term stability only when kept in MeOH, maintaining their structural integrity up to 3 years, as confirmed by consistent TEM imaging and DLS size evaluation (Figure S18). Upon dilution in H_2_O to concentrations comparable to those used for cellular administration, the framework rapidly disintegrated, confirming the intrinsic fragility of ZIF‐8 under biologically relevant conditions (Figure S19). This finding is extremely important, as it directly impacts the potential biomedical use of ZIF‐8 composites.

To address this limitation, solvent change and stabilization of the MOF with a PMA‐based amphiphilic polymer (poly[isobutylene‐alt‐maleic anhydride]‐graft‐dodecyl) coating protocol was adapted from a previous reported protocol on Au‐MOF core‐shell structures (Figure 5A) [54]. After PMA functionalization and redispersion in H_2_O, the purified composites retained the cubic morphology and size for at least 4 weeks (Figure 5B), showing a monomodal hydrodynamic peak as measured by DLS, with a PdI below 0.2 as an indication of their good dispersion in H_2_O (Table S6).

**FIGURE 5:**
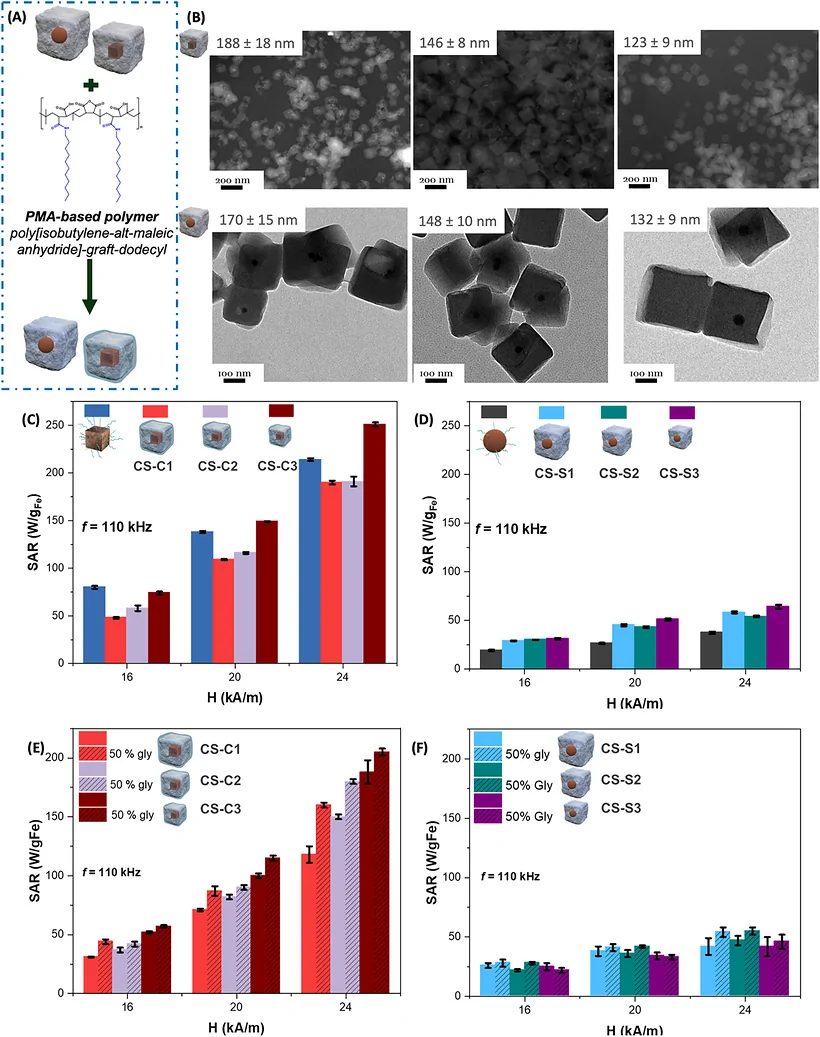
(A) Scheme of the PMA coating of CS composite, and (B) SEM and TEM images of the CS after water transfer with PMA; Comparative SAR values for (C) Fe_3_O_4_ (cubes, 18 nm), and (D) Fe_3_O_4_ (spheres, 22 nm) and their CS at different sizes measured by AC magnetometry at *f* = 110 kHz and *H* = 16, 20, and 24 kA m^−1^. All measurements were performed using 40 µL of sample at a concentration of 1 g L^−1^ Fe; SAR media viscosity dependence measurements under AMF at *f* = 110 kHz and *H* = 16, 20, and 24 kA m^−1^ of (E) CS having cubic core, and (F) NCs having spherical core. The percentages of glycerol are 0% and 50% in a water solution at 0.5 mg mL^−1^ Fe.

To evaluate the heating performances of the CS composites, once coated with PMA, the SAR values were determined by inductive measurements using AC magnetometry at different field intensities and frequency (Figure 5C,D). The evaluation of SAR performance at clinical frequencies (110 kHz, 16 KA m^−1^) showed a clear shape‐dependent effect, with the cubic–shaped composites displaying higher heating efficiency compared with the spherical counterparts (Table S7). Indeed, higher SAR values were obtained on the cubic‐shaped composites when increasing the field to 24 kA m^−1^, obtaining values of 250 ± 1 W/gFe when decreasing the ZIF‐8 shell thickness on the NPs surface to 123 ± 1 nm (CS‐C3). The spherical‐shaped composites (CS‐S1‐3) follow a similar trend, but smaller SAR values are obtained, reaching 55 ± 1 W/gFe for the smallest ZIF‐8 thickness (CS‐S3, 132 nm) in the same conditions. The heating performance of the ZIF‐8 composites exhibit similar SAR values (74 ± 1 W/gFe) with respect to the CTAB‐coated Fe_3_O_4_ (80 ± 1 W/gFe), while when using the spherical core, the SAR values of the composite are slightly higher (31 ± 1 W/gFe) than the CTAB‐coated (19 ± 1 W/gFe) at the smallest shell thickness (123 and 132 nm respectively). This enhancement is attributed to the reduced inter‐particle interactions as ZIF‐8 embeds individual nanoparticles within the MOF matrix, whereas CTAB‐coated NPs tend to aggregate, limiting the movement of individual NPs and decreasing the heat generation efficiency.

To better understand the response of the CS in biological environment, their SAR characterization was also investigated under viscous solution conditions. For this purpose, the NPs were dispersed in aqueous solution with different percentages of glycerol (0% and 50%) at the concentration of 0.5 g L^−1^ Fe (Figure 5E,F). When comparing the SAR values of CS with spherical and cubic cores, independently of the magnetic core, these values were not decreasing with respect to the values in water, indicating a viscous‐independent heat capacity, and in some conditions were slightly increased in glycerol. For instance, in the case of the cubic core with a bigger ZIF‐8 shell (188 nm), the SAR increased from 31–118 W/gFe to 44–160 W/gFe at clinically relevant AMF conditions (110 kHz and 16–24 kA m^−1^). While understanding the origin of this phenomenon is challenging, it is possible that the magnetic composites could align during the SAR measurements, resulting in the observed increase, as previously reported [55, 56].

### 2.6 Loading of Doxorubicin Drug within Fe_3_O_4_@ZIF‐8 Core‐Shell Composite

To evaluate the loading ability of the synthesized composite, Doxo was selected as an FDA‐approved chemotherapeutic agent used in the treatment of brain, breast, and ovarian cancers (Figure S20 and Table S8) [57, 58, 59]. The encapsulation of Doxo within ZIF‐8 follows two different approaches: in‐situ and post‐synthesis encapsulation (Figure 6A). For the in‐situ encapsulation, CS‐Doxo(*i*) was synthesized by a two‐step procedure in which Doxo, at the desired concentration, was added to the aqueous zinc solution, keeping the Zn^2+^ concentration the same as in the synthesis of the empty MOF. Then, the procedure followed the same steps as the approach previously developed, adding the solution over the organic linker and followed by the CTAB‐coated Fe_3_O_4_ addition (Figure 6A, case 1). In the case of the post‐synthesis encapsulation, CS‐Doxo(*a*) in the desired concentration in MeOH was introduced to the already formed composite and mixed overnight (Figure 6A, case 2).

**FIGURE 6:**
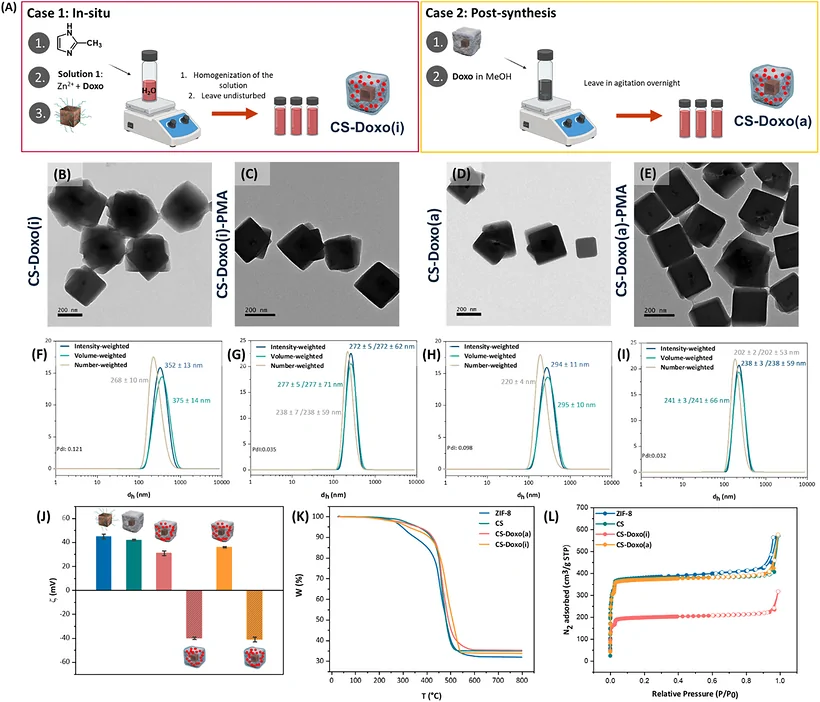
(A) Schematic illustration of the Case 1 – in‐situ encapsulation; and Case 2 – post‐synthesis encapsulation of Doxo; TEM images of (B) CS‐Doxo(*i*), (C) CS‐Doxo(*i*)‐PMA, (D) CS‐Doxo(*a*), and (E) CS‐Doxo(*a*)‐PMA; (F–I) Their respective DLS measurements; (J) Zeta‐potential of Fe_3_O_4_@CTAB, CS, CS‐Doxo(*i*), CS‐Doxo(*a*), and the PMA coated CS; (K) TGA analysis; and (L) N_2_ adsorption isotherms for ZIF‐8, CS, CS‐Doxo(*i*) and CS‐Doxo(*a*).

For both approaches, different initial Doxo concentrations (150, 250, and 500 µg mL^−1^) were screened to determine the best encapsulation efficiency (EE%) and loading efficiency (LE%). EE% was defined as the difference between the initial drug amount and the residual drug in the supernatant, normalized to the initial drug amount. LE% was defined as the ratio between the amount of Doxo (mg) and the amount of MOF composite, expressed as mg of Zn. EE% values were calculated by interpolating the fluorescence intensity of the initial Doxo solution and that of the supernatants collected after centrifugation and washing against a Doxo calibration curve. For the in‐situ approach, the first wash (H_2_O) and second wash (MeOH) were analyzed, while for the post‐synthesis approach, the first MeOH wash was used (Figure S21; Tables S9 and S10). The rationale behind this choice for the two different samples is related with the distinct loading strategies employed: in the in‐situ method, Doxo is mixed with ZIF precursors in water, during ZIF‐8 crystallization, and subsequent aqueous to MeOH exchange solvent is required to preserve the structural integrity of the composite. For this reason, both H_2_O and MeOH washings are recovered and measured to evaluate the unloaded Doxo. Whereas in the post‐synthesis method, Doxo is introduced over a pre‐formed MeOH stable framework, for which a single MeOH wash is sufficient to remove non‐specifically adsorbed Doxo. Based on this, for the in‐situ composites, EE% values of 96%, 98%, and 82% were achieved, corresponding to 27%, 39%, and 41% LE% values for 150, 250, and 500 µg mL^−1^ of Doxo, respectively (Table 3 and Figure S22A–C). In contrast, the post‐synthesis approach (Table 3 and Figure S22D–F) results in lower EE% values of 80%, 79%, and 80% corresponding to 0.12, 0.11, and 0.14 mg Doxo/mg Zn for 150, 250, and 500 µg mL^−1^ of Doxo, respectively. These values are significantly higher than previously reported with Doxo, where loading efficiencies of 10% or 25% were obtained using a similar in‐situ encapsulation method [60], and 12% when using post‐synthesis approach [30].

**TABLE 3:** Drug loading for CS composite. Nominal concentration of drug, drug amount calculated from UV–Vis, percentage of encapsulation efficiency (EE%), mg Drug/ mg Zn, and loading efficiency (LE%).

Encapsulation	Drug	[Drug] µg/mL	[Drug] µg/mL IN	EE%	mg drug/mg Zn	LE%
*In‐situ*	Doxo	150	144	96	0.27	27
		250	244	98	0.39	39
		500	410	82	0.41	41
*Post‐synthesis*		150	120	80	0.12	12
		250	198	79	0.11	11
		500	295	59	0.14	14

Furthermore, the structural characterization by TEM imaging of CS‐Doxo(*i*) shows that the composite retains the typical cubic‐shape morphology of the magnetic‐MOF unloaded with Doxo up to a Doxo‐loaded concentration of 250 µg mL^−1^ (nominal concentration) (Figure S22A–C). Instead, when using an encapsulating solution of 500 µg mL^−1^ of Doxo (nominal concentration), the loss of the perfect cubic shape and the presence of voids and mesopore structures were observed. Likely, under this condition, the high amount of Doxo interferes with the crystal growth of ZIF‐8 structure. This observation aligns with previous findings in the literature when using high concentrations of Doxo on MOF of ZIF‐8 composition [60]. The high loading capacity and the structural difference are likely ascribed to the coordination bonds between Doxo and the Zn^2+^ cations [60], rather than being confined within the pores of the ZIF‐8 [31, 60]. The largest pore size of the ZIF‐8 structure has a diameter of 11.6 Å interconnected by 6‐ring windows of a diameter of 3.4 Å, smaller than Doxo's dimensions of 14.6 × 1 0.2 × 6.9 Å, preventing diffusion through the framework [29]. In the insitu approach, Doxo remains entrapped in the structure as the ZIF‐8 assembly, binding with the Zn^2+^ cations, replacing some of the imidazole ligands, and stabilizing its incorporation. On the contrary, the post‐synthesis loading methodology preserves the core‐shell structure of the CS‐Doxo(*a*) across all Doxo nominal concentrations, without altering its morphology (Figure S22D–F). These results are indeed expected since Doxo adsorption occurs at the surface of the pre‐formed MOF. As Vasconcellos et al.  [31] reported, Doxo interacts with the Zn^2+^ cations via chelating sites on the quinone and the phenolic ring oxygens on both sites of the anthracycline aromantic moieties. In the ZIF‐8, Zn^2+^ cations exhibit tetrahedral geometry coordinated by four imidazole groups; however, Zn^2+^ cations on the surface may become partially solvated, generating more accessible coordination sites. Under these conditions, Doxo can bind to the Zn^2+^ available on the surface, while maintaining the tetrahedral coordination geometry of the metal center. Also in this case, the composites were PMA‐coated and redispersed in H_2_O, retaining the cubic morphology and size for at least 4 weeks (Figure 6B–E), showing a monomodal hydrodynamic peak as measured by DLS, with a PdI below 0.2 as an indication of their good dispersion in H_2_O (Figure 6F–I). Moreover, the zeta‐potential of all CS composites remains positive upon Doxo encapsulation (Figure 6J).

Furthermore, TGA performed under air atmosphere (Figure 6K) showed a sharp weight loss (60 wt.%) at around 400°C, associated with the total combustion of MeIM and the formation of different inorganic residues: ZnO in the case of ZIF‐8 or ZnO and Fe_3_O_4_ in the case of composites.

N_2_ adsorption‐desorption measurements were used to evaluate whether Doxo encapsulation alters the porosity of the composites. As illustrated in Figure 6L, in all cases, the type I isotherm of ZIF‐8 was preserved, confirming retention of microporosity [51]. However, in the in‐situ loaded samples, there is a reduction in uptake, decreasing from 387 cm^3^/g to 214 cm^3^/g, consistent with the steric hindrance created by the large size of Doxo molecules. This not only can block some of the pore entrances but also reduces the available pore volume for adsorption [38]. In the case of the CS with Doxo encapsulated post‐synthesis, no change with respect to the empty CS can be observed, probably because, as explained before, Doxo remains on the surface coordinating with the Zn^2+^ ions linking to the MOF, rather than being confined within its pores (see Table S11 and Figures S23–S25).

The stability of the Doxo‐loaded CS was studied in water by repeated DLS measurements of the hydrated size, the d_h_,_I_ over a period of three months (Figure 7A). The samples remained stable up to three weeks as seen by the maintenance of the d_h_ values; while on the long‐term, after 3 months, there was an increase of the d_h_ size from 279 ± 4 nm to 431 ± 172 nm for in‐situ Doxo‐loaded CSs (CS‐Doxo(*i*)‐PMA) and from 298 ± 5 to 423 ± 181 nm for post‐synthesis Doxo‐loaded composite (CS‐Doxo(*a*)‐PMA). The PdI also showed an increase from 0.059 to 0.6 for CS‐Doxo(*i*)‐PMA and from 0.098 to 0.728 for CS‐Doxo(*a*)‐PMA, meaning that the composites were starting to get more polydisperse as an indication of possible structural transformation due to breakage, fragmentation, and aggregation. The change in morphology after three months was also checked via TEM analysis (Figure 7B). For both CSs composites, a clear change in structural integrity with breakage of the structures with respect to the freshly prepared samples was evident. However, it was observed that the in‐situ Doxo‐loaded composite presented a faster degradation with respect to the post‐synthesis Doxo‐loaded sample. This could be due to the easier degradation of the CS, thus leading to breakage of the coordination bonds between Doxo and the linker part in the ZIF‐8 structure. The higher instability observed for the CS‐Doxo(*i*)‐PMA in aqueous media can be attributed to the role of Doxo during ZIF‐8 shell formation. In this case, Doxo is present during nucleation and growth of the framework and can interact with Zn^2+^ through coordination with quinone and phenolic oxygen groups, while Zn^2+^ simultaneously coordinates with MeIM linkers to construct the ZIF‐8 network, as previously reported [31]. In contrast to the strong and extended Zn‐imidazolate coordination bonds that define the MOF's structure, Zn‐Doxo interactions are weaker, reversible, and do not contribute to the framework connectivity. Thus, Doxo(*i*) acts as a competing coordination species during shell growth, increasing defect density, which is known to cause hydrolytic instability of ZIF‐8 frameworks. Consequently, the resulting defect‐rich frameworks are known to be more susceptible to Zn‐N bond cleavage in aqueous media, leading to accelerated degradation compared to post‐synthetically loaded systems, where Doxo adsorption occurs on a fully formed and structurally stabilized ZIF‐8 surface [29, 61, 62]. PXRD analysis was also performed on both samples; however, no change in the ZIF‐8 crystallinity has been observed over the same time span of 3 months (Figure S26). To extend shelf life and improve long‐term stability, the composites were freeze‐dried, yielding powders that after re‐dispersing the sample in water remained structurally intact, as confirmed by consistent DLS size distributions and zeta‐potential values before and after storage (Figure S27).

**FIGURE 7:**
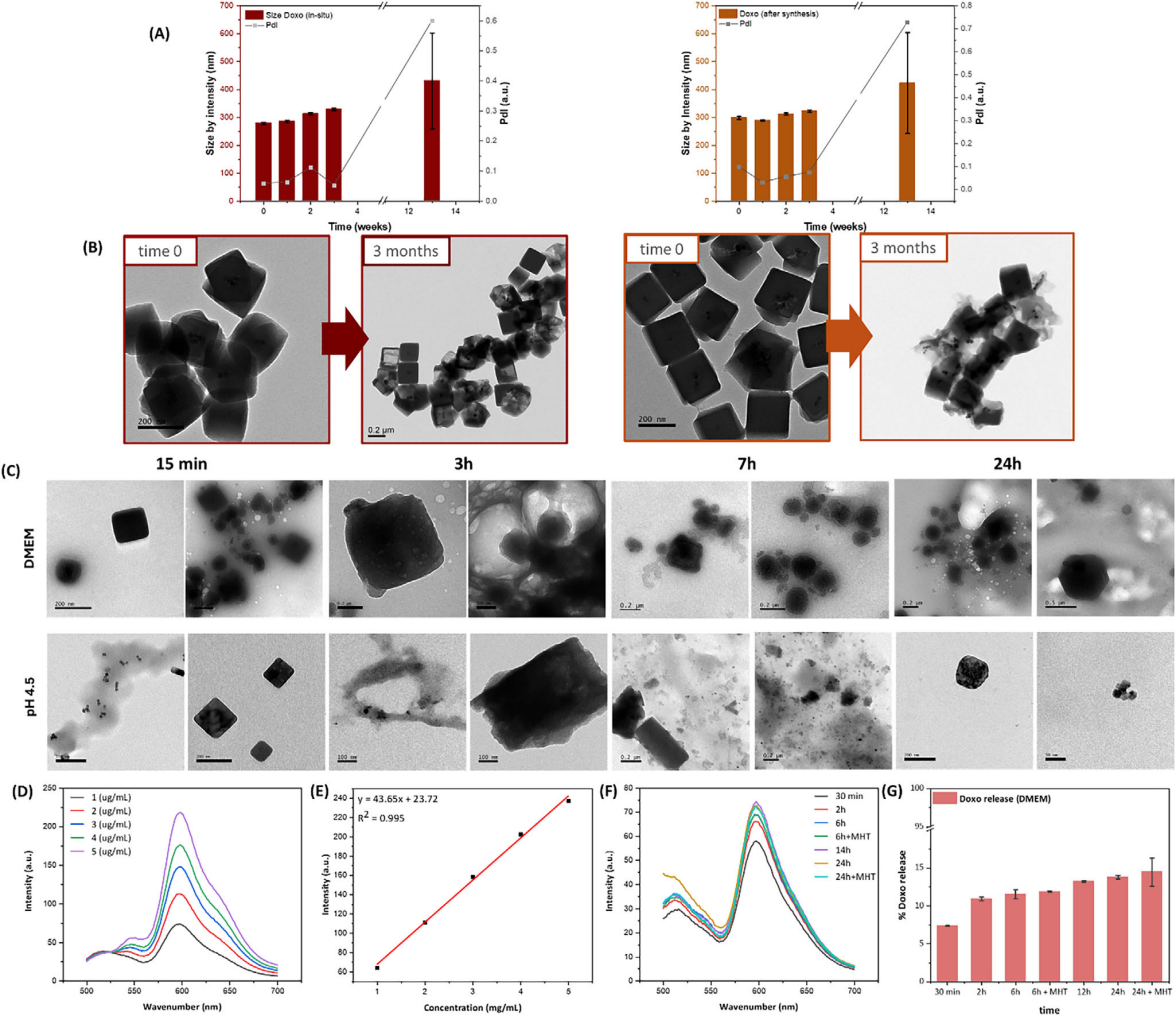
(A) Degradation over time by DLS of the Doxo‐loaded composites in‐situ (red) and post‐synthesis (brown). St. Dev. refers to the error between the measures; (B) TEM showing the degradation of the composites loaded with Doxo in‐situ (red) and post‐synthesis (brown); (C) TEM images of the CS‐Doxo(*i*)‐PMA in different conditions: DMEM, and PBS pH 4.5, over 24 h of incubation Fluorescence spectrum of Doxo and its corresponding calibration curve: in DMEM (D‐E); (F) Fluorescence spectrum of CS‐Doxo(*i*)‐PMA in DMEM at different time points and upon AMF application; and (G) Release % profile of Doxo from CS‐Doxo(*i*)‐PMA at the different time points: 30 min, 2, 6, and 6 h + MHT, 14, 24, and 24 h + MHT.

### 2.7 Doxo Release Study

Considering the faster degradation in water of the CS‐Doxo(*i*)‐PMA with respect to the CS‐Doxo(*a*)‐PMA, we have opted to study the stability in different media and the Doxo release only for the CS‐Doxo(*i*)‐PMA. Stability was assessed by dissolution of CS‐Doxo(*i*)‐PMA in different physiological media including complete DMEM (supplemented with 10% fetal bovine serum (heat‐inactivated FBS)) to simulate the nutrient‐rich environment that cells experience in vitro, NaOH (0.01 M) solution at pH = 9 to predict how MOF will react to basic pH, phosphate‐buffered saline (PBS) solution at pH = 7.5 to mimic the extracellular neutral pH in most tissues and organs, and PBS at pH = 4.5 to simulate the acidic environment found in lysosomes and TEM images were taken at different time points over a 24 h time (Figure 7C and Figure S28). The composites in complete DMEM appear to be much more stable than in the other media, since after 24 h, their morphology was still recognizable, although the appearance of some subproducts is already visible. Under alkaline (NaOH 0.01 M) and slightly basic (PBS at pH 7.5) conditions, the CSs' morphology was more severely compromised, with evidence of uncoated NPs becoming apparent (Figure S28). In comparison, the degradation at pH 4.5 is accelerated, and more tiny structures are present after just a few minutes (Figure 7C).

Due to the better stability in DMEM of the CS‐Doxo(*i*)‐PMA, Doxo release studies were conducted on this sample by diluting the composites at (15 mgZn L^−1^) in complete media and quantifying the Doxo release by fluorescence spectroscopy (Figure 7D–F). Less than 15% of Doxo was released from the CS‐Doxo(*i*)‐PMA after 24 h as non‐specific release (Figure 7G).

Considering the reported results, we can conclude that the stability of the Doxo within the CS‐Doxo(*i*)‐PMA was sufficiently high to enable us to perform uptake and release studies on cells. Moreover, we would expect the release of approximately 14% of Doxo from the CS in the cell experiments.

To understand the magnetic response of the CS‐Doxo(*i*)‐PMA CS in a biological or viscous environment, as the one in cells, the SAR was evaluated in aqueous solution at different percentages of glycerol (0% and 50%) at concentration of 0.5 g L^−1^ of CSs. At clinically relevant AMF conditions (110 kHz and 16–24 kA m^−1^), the SAR values of the CS‐Doxo(*i*)‐PMA CSs seemed to increase in the glycerol from 31–118 W/gFe to 44–160 W/gFe at clinically relevant AMF conditions (110 kHz and 16–24 kA m^−1^) (Figure S29). Instead, the SAR values in DMEM decreased in values to 21–89 W/gFe with respect to the CS dispersed in H_2_O and glycerol, from 31–118 W/gFe and 44–160 W/gFe, respectively.

### 2.8 Cell Studies

#### 2.8.1 Cytotoxicity Tests

To move forward with the in vitro studies, we have selected CS‐Doxo(*i*)‐PMA composite rather than CS‐Doxo(*a*)‐PMA because of its highest drug loading efficiency and the fast degradation rate (Table 3).

First, to access the cytotoxicity of CS‐PMA (without Doxo) and CS‐Doxo(*i*)‐PMA (with Doxo) composites, an in vitro MTT assay was performed on U‐87 MG glioblastoma and MDA‐MB‐468 breast cancer cells. U‐87 MG cells (8 × 10^3^ cells/well, 96‐well plate), and MDA‐MB‐468 cells (1.5 × 10^4^ cells well^−1^, 96‐well plate) cultured in 2D, were incubated with CS‐PMA, and CS‐Doxo(i)‐PMA at different concentrations (1, 2, 4, and 8 mgZn L^−1^ in a total volume of 100 µL complete DMEM) for continuous NPs exposure at different time points (24, 48, and 72 h). It is worth noting that we have decided to base our studies on the Zn concentration, since it is the most abundant species in our materials; therefore, it is easier to assess the concentration by ICP. Moreover, Zn is more likely to induce toxic effects with respect to Fe (Table S12) [28]. CS‐PMA exhibited time‐dependent and dose‐dependent toxicity in both U‐87 MG cells (Figure 8A,B) and MDA‐MB‐468 (Figure S30A,B). When incubating with lower CS‐PMA composite doses, at 2 mg Zn L^−1^, the viability remained above 80% for both cell lines through 72 h of continuous incubation.

**FIGURE 8:**
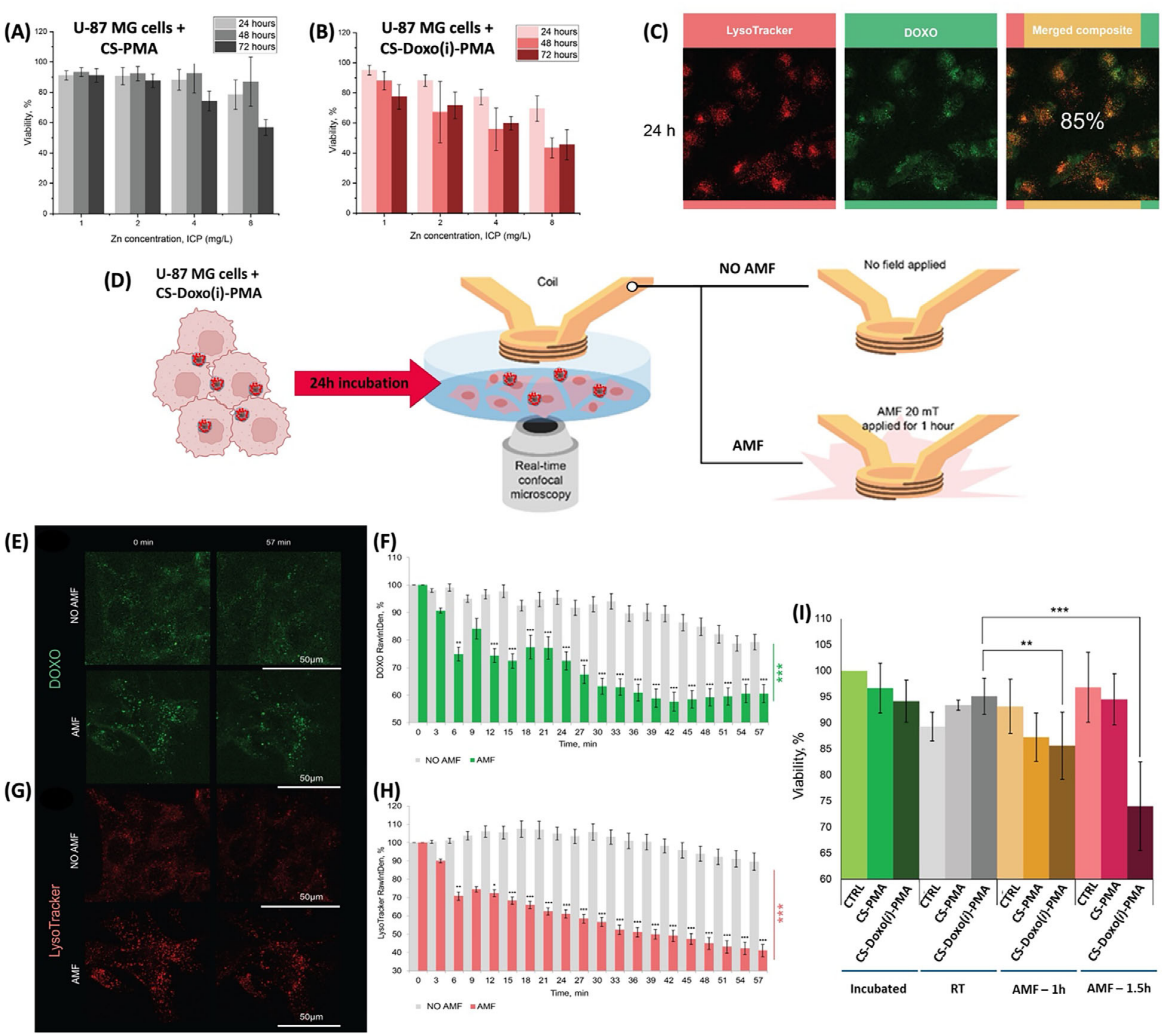
Cell viability of U‐87 MG cells after 24, 48, and 72 h of incubation with the range of concentrations of CS‐PMA (*n* ≥3) (A) and CS‐Doxo(*i*)‐PMA (*n*≥3) (B); (C) Lysosomal localization by confocal imaging of CS‐Doxo(*i*)‐PMA composite after 24 h of incubation with U‐87; (D) Schematic representation of the experimental setup for real‐time observation under AMF application. First, the cells are incubated with 2 mgZn L^−1^ of CS‐Doxo(*i*)‐PMA. After 24 h incubation, half of the samples were exposed to the AMF for 1 h, another half was left under confocal with no field on. In both cases, the confocal z‐stacks were recorded every 3 min; (E) Doxo‐associated fluorescence at 0 min and 57 min with and without AMF exposure; (F) Doxo fluorescence intensity recorded over 1 h with AMF (green) and without field application (grey); (G) LysoTracker Deep Red fluorescence at 0 min and 57 min with and without AMF exposure; (H) Change recorded in LysoTracker fluorescence over 1 h with AMF (red) and without field application (grey). Statistical analysis was done with Kruskal‐Wallis and Dunn's multiple comparison test for the fluorescence intensity values, time‐dependent change (black symbols), and with Mann‐Whitney nonparametric test for overall trends (red/green symbols); *p* value 0.033 (*); 0.002 (**); < 0.001 (***). For each group, *n* ≥2 independent experiments were held with the total number of 50 – 120 cells analyzed per condition. (I) U‐87 MG cell viability in Ctrl—complete DMEM; 2 mgZn L^−1^ CS‐PMA, 2 mgZn L^−1^ CS‐Doxo(*i*)‐PMA. Incubated for 1.5 h—“Incubated”; exposed to RT for 1.5 h—“RT”; exposed to 1 h of AMF—“AMF – 1 h”; exposed to 1.5 h of AMF—“AMF – 1.5 h” (*n* ≥ 3). Statistical analysis: Brown‐Forsythe and Welch ANOVA tests. Dunnett T3 multiple comparison correction; *p* value reporting style is NEJM: 0.12 (ns); 0.033 (*); 0.002 (**); < 0.001 (***).

At 4 mgZn L^−1^, the relative cell viability on U‐87 MG was affected significantly only after 72 h of incubation, reaching 74 ± 6% of control cells' viability. Incubation of U‐87 with CS‐PMA at higher concentrations (8 mgZn L^−1^) results in a viability drop to approximately 57 ± 5% upon 72 h of incubation, compared to control cells (Figure 8A). In turn, co‐incubation of MDA‐MB‐468 with CS‐PMA significantly decreases the cell viability to 72 ± 12% at 4 mgZn L^−1^ and 48 h of incubation (Figure S30A). Therefore, the concentration of the CS‐PMA composite of 2 mgZn L^−1^ given the non‐toxic profile even at 72 h post incubation (Figure 8A), was chosen for further in vitro experiments.

Incubation with CS‐Doxo(*i*)‐PMA composite shows also time and dose dependent toxicity. At 2 mgZn L^−1^, corresponding to 0.028 µg mL^−1^ of Doxo associated, no cytotoxicity was observed at 24 h (viability above 80%), with a slight increase at 48 h and 72 h post incubation indicating a good encapsulation effect and a slow nonspecific release toxicity (Figure 8B).

Instead at 4 mgZn L^−1^ (0.056 µg mL^−1^ Doxo) viability drop in U‐87 MG cells to approximately 56 ± 14% at 48 h, (Figure 8B). High cytotoxicity on U‐87 MG cells, below 50%, is reached upon incubation with 8 mgZn L^−1^ of CS‐Doxo(*i*)‐PMA (0.112 µg mL^−1^ Doxo) composite for longer than 48 h. Similar cytotoxic dose dependent trend was also observed for MDA‐MB‐468 cell line; CS‐Doxo(*i*)‐PMA displays high cytotoxicity, inhibiting the cell viability by 45 ± 5% after 72 h of incubation with 8 mgZn L^−1^ of CS‐Doxo(*i*)‐PMA (Figure S30B). This cytotoxic effect of Doxo‐loaded composite is likely caused by the non‐specific drug release (Figure 8A,B).

#### 2.8.2 Cellular Uptake and Subcellular Localization

The uptake kinetics was quantified on the U‐87 MG cell line using the Doxo‐related fluorescence signal of CS‐Doxo(*i*)‐PMA composites detected in cells by flow cytometry (FACS) after 2, 24, and 48 h of coincubation with 2 mgZn L^−1^ CS‐Doxo(*i*)‐PMA composite. The results were plotted based on the percentage of total cells exhibiting Doxo fluorescence (Figure S31A,B) and based on the Doxo mean fluorescence intensity (MFI) of cells up taking CS‐Doxo(*i*)‐PMA (Figure S31C,D). A clear increase in both the number of fluorescent cells and Doxo MFI was observed at 24 h (85 ± 4%, MFI = 4.0 ± 0.1%), which remains stable after 48 h (68 ± 25%, MFI = 4.0 ± 0.8%), compared to 2 h (56 ± 9%, MFI = 3.0 ± 0.1%) of incubation with CS‐Doxo(*i*)‐PMA. However, given the low cytotoxicity at 24 h (88 ± 4%) compared to 48 h (67 ± 20%), and the uptake kinetics, 24 h was chosen as the optimal co‐incubation time for CS‐Doxo(*i*)‐PMA composite before AMF application for the latter stage of the experiments.

The subcellular localization of CS‐Doxo(*i*)‐PMA composites in U‐87 MG was then studied in cells by confocal microscopy. 1 × 10^5^ U‐87 MG cells were incubated with CS‐Doxo(*i*)‐PMA composite (500 µL at 2 mgZn L^−1^ corresponding to 0.028 µg mL^−1^ of Doxo) for 24, 48, and 72 h. Prior to microscopy analysis, LysoTracker Deep Red (10 nM) was added to cells in order to stain the lysosomes, while Hoechst 33342 dye (10 nM) was used to stain the cell nuclei (Figure S32). After 24 h of incubation with U‐87 cells, the successful internalization of the CS‐Doxo(*i*)‐PMA can already be observed in the lysosomes (Figure 8C). That finding is in contrast to previous works, where similar composites remained in the cytoplasm [60], and unlike free Doxo, which tends to accumulate in the nuclei [63]. Given the hydrodynamic size of the CS‐Doxo(*i*)‐PMA (232 ± 6 nm), endocytosis followed by degradation in the lysosomes can be expected, which was previously shown for many other nanostructures of similar dimensions [64].

By quantifying the area of the overlay between the Doxo and LysoTacker fluorescence (represented with orange signal) (Figure S33), it is possible to estimate the percentage of the CS‐Doxo(*i*)‐PMA in the lysosomes. The overlapping area at 24, 48, and 72 h was calculated to be 85%, 86%, and 79% respectively (Figure 8C and Figure S34).

#### 2.8.3 Real‐time Observation of AMF Application

To study the effect of AMF on the release of Doxo from the composites, 2D monolayer cultured U‐87 MG cells were incubated with CS‐Doxo(*i*)‐PMA at 2 mgZn L^−1^ concentration and equivalent to 0.028 µg mL^−1^ of Doxo, for 24 h to maximize their cell uptake and to ensure no toxicity for the CS‐PMA and CS‐Doxo(*i*)‐PMA. Next, non‐internalized composites were removed by washing the cells fresh medium, and cells were stained with Lysotracker deep red (10 nM). The cells were exposed or not to AMF for 1 h at clinically safe AMF conditions (*H* = 16 kA m^−1^ (20 mT) and *f* = 300 kHz) using a home‐made miniaturized electromagnets allowing real‐time imaging by confocal microscopy (Figure 8D) [65]. The temperature of the cell incubation medium was monitored and maintained around 37°C during the experiment. The effect caused by AMF application was studied by analyzing both Doxo (green bars, Figure 8E,F) and LysoTracker (red bars, Figure 8G,H) fluorescence intensity (Figure S35). A clear time‐dependent drop in the fluorescence intensity values of Doxo (green bars, Figure 8F) up to 61 ± 3% (*p* < 0.001), and of LysoTracker (red bars, Figure 8H) up to 41 ± 3% (*p* < 0.001) was significantly observed when exposing the cells to AMF for 1 h. Doxo signal decrease is likely connected to the release of Doxo molecules from the lysosomes to the cytoplasm following the degradation of the MOF composite, that can be triggered by the pH in the lysosomes. Moreover, the decrease of Doxo fluorescent intensity was significantly detected since 6 min of AMF application, indicating that Doxo was early released by AMF exposure (Figure 8F). Significant loss of LysoTracker signal was also early and significantly detected since 6 min of AMF exposure, revealing the damage to the lysosomes induced by magnetic hyperthermia, which is most likely associated with the degradation of the composite and consequent release of Doxo. In contrast, when analyzing the Doxo (grey bars Figure 8F) and LysoTracker (grey bars Figure 8H) fluorescence intensity signals on cells in the absence of AMF exposure, no significant change was observed between the starting point and the endpoint. Since no bulk temperature rise was detected and no release occurred in cell‐free DMEM controls, the observed lysosomal disruption under AMF must originate from local effects on the composites once internalized, most plausibly nanoscale heating or mechanical oscillation within the lysosomal environment.

#### 2.8.4 Cell Proliferation after AMF Application

The AMF‐enhanced cytotoxicity was also confirmed by analyzing cell viability on both U‐87 MG and MDA‐MB‐468 cells, using the PrestoBlue assay, assessed 24 h post‐AMF treatment. All values were calculated based on the control sample, referred to as “Incubated—CTRL” that was left incubating at 37°C, 5% CO_2_, and 95% relative humidity in absence of CS. Notably, the *p* value obtained with 2‐way ANOVA exceeding 0.99 indicate that the difference between CTRL samples and CS‐PMA samples undergoing 1 or 1.5 h of hyperthermia are not statistically significant. The results showed that AMF exposure significantly decreased the viability of CS‐Doxo(*i*)‐PMA‐loaded U‐87 MG (86 ± 6%, *p* = 0.002), compared to the cells with CS‐Doxo(*i*)‐PMA left at RT without AMF application (95 ± 3%) (Figure 8I). However, the most significant decrease is provided by a combination of incubation with CS‐Doxo(*i*)‐PMA and 1.5 h of AMF application, which resulted in a viability drop to 74 ± 8% (*p* < 0.001) (Figure 8I). Moreover, in vitro drug release experiments performed in similar conditions (complete DMEM, and preincubated at 37°C for 24 h), yielded approximately 0.1 µg mL^−1^ of Doxo, matching the nominal concentration of the CS used for the cell study (2 mgZn L^−1^). The incubation of this amount of free Doxo for 24 h decreased the cell viability of U‐87 MG cells to 74 ± 8%, which is very close to the cell viability inhibition induced by CS‐Doxo(*i*)‐PMA (2 mgZn L^−1^) and 1.5 h of AMF application (Figure S36). Finally, we also showed that 1.5 h AMF exposure also reduced the viability of CS‐Doxo(*i*)‐PMA, providing MDA‐MB‐468 cells (82 ± 7%) compared to cells subjected to the same composite, but not exposed to AMF (97 ± 6%, RT) (*p* = 0.002) (Figure S37). Altogether, these results, accompanied by the fluorescent signal decrease of Doxo and LysoTracker (Figure 8F,H), strongly suggest that AMF induced Doxo leakage and lysosomes permeabilization leading to cytotoxicity.

## 3 Conclusions

In this work, we introduced a facile seeded growth method for the synthesis of two core‐shell composites, featuring Fe_3_O_4_ in spherical and cubical shapes as the magnetic core, and ZIF‐8 as the shell. This approach enables precise control over the ZIF‐8 shell thickness by adjusting the CTAB amount used for Fe_3_O_4_ coating during the MOF growth. The CS composites exhibit magnetic properties affected by the ZIF‐8 shell and the core geometry. In both cases, the thinner shell exhibited enhanced magnetic properties. Furthermore, it was found that the magnetic response of the NCs is determined by the thickness of the shell, with the thinner shell having enhanced heating properties. These CSs not only preserve their heating performance but also exhibit impressive encapsulation and loading efficiencies when introduced within ZIF‐8 by the in‐situ approach. The Doxo‐loaded composites exhibited high stability in DMEM and showed promising performance for alternating high‐frequency magnetic field (AMF)‐triggered release. In vitro experiments using U‐87 MG glioblastoma cells and MDA‐MB‐468 breast cancer cells indicated that the composites are non‐cytotoxic at concentrations up to 2 mgZn L^−1^ and are effectively internalized by the cells. Most composites were localized within the lysosomes. Furthermore, the potential of using Doxo‐loaded composites for magnetically induced hyperthermia (MHT) was explored. Although the application of an AMF did not immediately trigger Doxo release in complete DMEM, it significantly increased lysosomal permeabilization, thus contributing to Doxo lysosomal escape. Continuous exposure to AMF for 1 h or 1.5 h resulted in subsequent leakage of Doxo, leading to enhanced cytotoxicity. Altogether, these findings demonstrate that the Fe_3_O_4_@ZIF‐8 core–shell composites act as efficient intracellular nanocarriers, in which AMF exposure induces lysosomal permeabilization, promotes Doxo release and escape upon ZIF‐8 degradation at acidic pH, and significantly enhances cytotoxicity, establishing a proof‐of‐concept for AMF‐triggered drug delivery in cancer cells.

## Conflicts of Interest

The authors declare no conflicts of interest.

## Supporting information




**Supporting File 1**: smll74972‐sup‐0001‐SuppMat.docx.

**Supporting File 2**: smll74972‐sup‐0002‐VideoS1.mp4.

## Data Availability

The data that support the findings of this study are available from the corresponding author upon reasonable request.
