# Supplementary material for: Seeded‐Growth of ZIF‐8 onto Magnetic Nanoparticles: Smart Nanocarriers with Ultrahigh Drug Loading for AMF‐Triggered Release
Source: Small. 2026 Aug 10;22(55):e74972. doi: 10.1002/smll.74972 (PMC13630407; doi:10.1002/smll.74972)
Supplement: Supplementary file 1 [file SMLL-22-e74972-s002.docx]

**SUPPORTING INFORMATION**

**Seeded-Growth of ZIF-8 onto Magnetic Nanoparticles: Smart Nanocarriers with Ultrahigh Drug Loading for AMF-Triggered Release**

Ana Maria Panaite,^a,b^ Aleksandra Predeina,^a^ Aitor Alvarez Lorenzo,^c^ Manuel Ceballos,^c^ Pascal Clerc,^d^ Niccolò Silvestri,^a^ Andrea Griesi,^a^ Giorgio Divitini,^a^ Julian Carrey,^e^ Veronique Gigoux,^d^ Pablo del Pino,^c*^ Teresa Pellegrino^a,*^

^a^Istituto Italiano di Tecnologia (IIT), via Morego 30, 16163 Genoa, Italy

^b^Universita degli Studi di Genova (UniGe), via Dodecanaso 31, 16146, Genoa, Italy

^c^Centro Singular de Investigación en Química Biolóxica e Materiales Moleculares (CiQUS), Departamento de Física de las Partículas, Universidade de Santiago de Compostela, 15782, Santiago de Compostela, Spain

^d^CRCT, Université de Toulouse, Inserm U1037, CNRS, Université Toulouse III-Paul Sabatier, Centre de Recherches en Cancérologie de Toulouse, Toulouse, France

^e^Laboratoire de Physique et Chimie des Nano-Objets (LPCNO), INSA, CNRS UMR5215, Toulouse, France

*Corresponding authors: [pablo.delpino@usc.es](mailto:pablo.delpino@usc.es), [teresa.pellegrino@iit.it](mailto:teresa.pellegrino@iit.it)

**S1: Synthesis of composites - Supplementary figures**

**Figure S1**: Comparison of two MNPs samples having different shapes: iron oxide nanocubes and nanospheres at the same magnetic volume. TEM images and size distribution of (A, E) cubic Fe_3_O_4_ and (B, F) spherical Fe_3_O_4_, as synthesized; and after CTAB coating (C, G) cubic Fe_3_O_4_@CTAB and (D, H) spherical Fe_3_O_4_@CTAB. (A-D) shows TEM images; (E-H) shows the corresponding size distributions. (I, J) Hydrodynamic size distributions (Number-weighted) of cubic and spherical Fe_3_O_4_@CTAB, respectively. (K) Zeta-potential values measured after the water transfer protocol. The pH was not adjusted to a specific value. (L) FT-IR spectra of spherical Fe_3_O_4_@CTAB (**black line**), cubic Fe_3_O_4_@CTAB (**blue line**), CTAB ligand (**red line**), and oleic acid (**orange line**). Important peaks are indicated by the shaded regions.

**Figure S2**: (A) Image of reactions using different amount of CTAB (left 0.2M, middle 0.1M and right 0.05M) as well as magnetic stirrer; (B) DLS of the sample after washing, in which was used 0.05M CTAB; (C) Image of reaction taking place in a sonication bath at 60 °C; (D) DLS of the sample after washing. Hydrodynamic diameters (d_h_) (mean value ± standard deviation SD from 3 measurements).

**Table S1:** Polydispersity index (PdI) and Hydrodynamic diameters (d_h_) (mean value ± standard deviation SD from 3 measurements) as derived from DLS measurements of CTAB-coated cubic Fe_3_O_4_ (18 ± 1 nm) and spherical Fe_3_O_4_ (22 ± 1 nm). d_h,I_ , d_h,V_ and d_h,N_ refer to the mean average hydrodynamic diameter from intensity-, volume-, and number-weighted DLS distributions, respectively. Zeta-potential values measured after the water transfer protocol. The pH was not adjusted to a specific value.

| Sample | PdI | d_h,I_ (nm) | d_h,V_ (nm) | d_h,N_ (nm) | Zeta-potential (nm) |
| --- | --- | --- | --- | --- | --- |
| **Fe_3_O_4_ (cube)** | 0.285 | 53 ± 10 | 28 ± 11 | 41 ± 4 | 45 ± 2 |
| **Fe_3_O_4_ (sphere)** | 0.149 | 109 ± 2 | 81 ± 3 | 55 ± 5 | 31 ± 2 |

**Figure S3**: SEM images of CS formation when (A) MeIM is added over the Zn^2+^ to start the reaction and (B) when Zn^2+^ is added over the MeIM to start the reaction; (C) DLS graphs showing the hydrodynamic sizes by intensity-weighted (d_h,I_).

**Table S2**: Table showing PdI and hydrodynamic diameters (d_h_) (mean value ± standard deviation SD) as derived from DLS measurements of CS.

| Sample | PdI | d_h,I_ (nm) | d_h,N_ (nm) |
| --- | --- | --- | --- |
| **CS (Case A)** | 0.099 | 670 ± 12 | 617 ± 25 |
| **CS (Case B)** | 0.176 | 313 ± 16 | 237 ± 7 |

**Figure S4**: Overview of STEM-EDS spectra collected for 216 nm CS. These spectra have been used for the quantification reported in **Table S3**.

**Table S3**: Elemental analysis by STEM-EDS obtained for CS composite with an average size of 216 nm.

|  | Mass Norm. [%] | Mass Error [%] | Atom [%] | Atomic Error [%] |
| --- | --- | --- | --- | --- |
| **Carbon** | 59.50 | 2.36 | 76.70 | 2.18 |
| **Zinc** | 22.80 | 2.42 | 5.40 | 0.69 |
| **Iron** | 0.62 | 0.09 | 0.72 | 0.02 |
| **Oxygen** | 4.30 | 0.41 | 4.16 | 0.81 |

**Figure S5**: Pawley refinement of PXRD pattern on the CS in MeOH compared to Fe_3_O_4_ and ZIF-8.

**Figure S6**: Comparative SAR values for Fe_3_O_4_ (cubes, 18 nm), and Fe_3_O_4_ (spheres, 22 nm) measured by AC magnetometry at (A) f = 110 kHz and H = 16, 20, and 24 kA/m and (B) f = 200 kHz and H = 16, 20, and 24 kA/m.

**Table S4**: SAR values measured by AC magnetometry at f =110 and 200 kHz, and H = 16, 20, and 24 kA/m for Fe_3_O_4_@CTAB (cube, 18 nm) and Fe_3_O_4_@CTAB (sphere, 22 nm).

| Sample | SAR (W/gFe) | | | | | |
| --- | --- | --- | --- | --- | --- | --- |
|  | **110 kHz** | | | **200 kHz** | | |
|  | **16 kA/m** | **20 kA/m** | **24 kA/m** | **16 kA/m** | **20 kA/m** | **24 kA/m** |
| Fe_3_O_4_@CTAB  (cube, 18 nm) | 80 ± 2 | 138 ± 1 | 214 ± 1 | 139 ± 3 | 262 ± 5 | 451 ± 5 |
| Fe_3_O_4_@CTAB  (sphere, 22 nm) | 19 ± 1 | 26 ± 1 | 37 ± 1 | 41 ± 2 | 76 ± 4 | 115 ± 5 |

**Figure S7**: Size distribution corresponding to **Figure 3**.

**Figure S8**: (A-C) DLS particle size distribution, XRD analysis, and RAMAN of ZIF-8 and CS with cubic cores; (D-F) same analysis for ZIF-8 and CS with spherical cores.

**Figure S9**: (A-B) TGA analysis, and N_2_ adsorption isotherms of ZIF-8 and CS with cubic cores; (C-D) same analysis for ZIF-8 and CS with spherical cores.

Recalculation of the inorganic and organic content (% wt = weight percentage) of the samples ZIF-8, CS-S1 (spheres, 170 nm), CS-S2 (spheres, 148 nm), CS-S3 (spheres, 132 nm), CS-C1 (cubes, 188 nm), CS-C2 (cubes, 146 nm), and CS-C3 (cubes, 123 nm), taking into account the evaporation and remaining solvents within the porosity T°<150 °C, is showing in the following (numerical values are provided in **Table S5**).

**Fe_3_O_4_ wt% in CS**: considering there is no loss of organic content of ZIF-8 (MeIM) during the synthesis:

$$Inorganic wt\% \mathrm{CS}_{\mathrm{equivalent}}=\frac{Organic wt \% CS}{Organic wt\% ZIF-8}\times Inorganic wt\% ZIF-8$$

$$\mathrm{Fe}_{3}O_{4} wt\%=Total inorganic wt\% CS-Inorganic wt\% \mathrm{CS}_{\mathrm{equivalent}}$$

**Table S5**: Organic and inorganic weight percentage (wt %) derived from TGA measurements in ZIF-8 and CS selected samples and estimated wt % of Fe_3_O_4_.

| Sample | Organic wt% | Inorganic wt% | Fe_3_O_4_ wt% |
| --- | --- | --- | --- |
| ZIF-8 | 68.1 | 31.9 | n/a |
| CS-S1 (spheres, 170 nm) | 66.4 | 33.6 | 2.6 |
| CS-S2 (spheres, 148 nm) | 66.2 | 33.8 | 2.8 |
| CS-S3 (spheres, 132 nm) | 67.9 | 32.1 | 0.3 |
| CS-C1 (cubes, 188 nm) | 66.2 | 33.8 | 2.8 |
| CS-C2 (cubes, 146 nm) | 67.1 | 33.0 | 1.6 |
| CS-C3 (cubes, 123 nm) | 71.4 | 28.6 | 3.3 |

**Figure S10**: BETSI analysis for ZIF-8.

**Figure S11**: BETSI analysis for CS-C1 (cubes, 188 nm).

**Figure S12**: BETSI analysis for CS-C2 (cubes, 146 nm).

**Figure S13**: BETSI analysis for CS-C3 (cubes, 123 nm).

**Figure S14**: BETSI analysis for CS-S1 (spheres, 170).

**Figure S15**: BETSI analysis for CS-S2 (spheres, 148 nm).

**Figure S16**: BETSI analysis for CS-S3 (spheres, 132 nm).

**Figure S17**: Magnetization vs applied magnetic field curves normalized to the Fe+Zn amount of spherical Fe_3_O_4_ and its composites; and of cubic Fe_3_O_4_ and its composites, and measured at 5 K.

**Figure S18**: Life shelf of CS in MeOH over time. TEM image of the Cs (A) at time 0 (just after synthesis), and (B) at time 3 years. (C) DLS graphs showing the hydrodynamic sizes by number-weighted (d_h,N_). Hydrodynamic diameters (d_h_) (mean value ± standard deviation SD / mean value ± FWHM).

**Figure S19**: TEM images of CS (A) with PMA coating, and (B) directly dissolved in H_2_O.

**Table S6**: PdI and hydrodynamic diameters (d_h,N_) (mean value ± standard deviation SD) as derived from DLS measurements of the CS before and after the PMA coating.

| Sample | Before PMA | | After PMA | |
| --- | --- | --- | --- | --- |
|  | d_h,N_ (nm) | PdI | d_h,N_ (nm) | PdI |
| CS-S1 (spheres, 170 nm) | 246 ± 6 | 0.022 | 247 ± 10 | 0.083 |
| CS-S2 (spheres, 148 nm) | 226 ± 6 | 0.017 | 221 ± 5 | 0.027 |
| CS-S3 (spheres, 132 nm) | 190 ± 6 | 0.035 | 192 ± 4 | 0.042 |
| CS-C1 (cubes, 188 nm) | 232 ± 6 | 0.024 | 237 ± 6 | 0.100 |
| CS-C2 (cubes, 146 nm) | 219 ± 8 | 0.016 | 215 ± 2 | 0.051 |
| CS-C3 (cubes, 123 nm) | 200 ± 9 | 0.005 | 201 ± 6 | 0.052 |

**Table S7**: SAR values measured by AC magnetometry at f = 110 kHz, and H = 16, 20, and 24 kA/m in water and 50% Gly for Fe_3_O_4_@CTAB (cube, 18 nm) and Fe_3_O_4_@CTAB (sphere, 22 nm) and their corresponding CS composites.

| Sample | SAR (W/gFe) in H_2_O | | | SAR (W/gFe) in 50% gly | | |
| --- | --- | --- | --- | --- | --- | --- |
|  | **110 kHz** | | | **110 kHz** | | |
|  | **16 kA/m** | **20 kA/m** | **24 kA/m** | **16 kA/m** | **20 kA/m** | **24 kA/m** |
| Fe_3_O_4_@CTAB  (cube, 18 nm) | 80 ± 1.5 | 138 ± 1.2 | 214 ± 1.4 | - | - | - |
| CS-C1 (188 nm) | 31 ± 0.4 | 71 ± 1.0 | 118 ± 7.0 | 44 ± 2.0 | 87 ± 4.0 | 160 ± 8.0 |
| CS-C2 (146 nm) | 37 ± 2.0 | 82 ± 2.0 | 150 ± 2.0 | 42 ± 2.0 | 90 ± 2.0 | 180 ± 2.0 |
| CS-C3 (123 nm) | 52 ± 1.0 | 100 ± 2.0 | 188 ± 10.0 | 57 ± 1.0 | 115 ± 2.0 | 205 ± 3.0 |
| Fe_3_O_4_@CTAB  (sphere, 22 nm) | 19 ± 0.8 | 26 ± 0.7 | 37 ± 1.1 | - | - | - |
| CS-S1 (170 nm) | 26 ± 2.0 | 38 ± 4.0 | 42 ± 7.0 | 28 ± 3.0 | 41 ± 3.0 | 54 ± 4.0 |
| CS-S2 (148 nm) | 22 ± 1.0 | 36 ± 3.0 | 47 ± 4.0 | 28 ± 1.0 | 42 ± 1.0 | 55 ± 3.0 |
| CS-S3 (132 nm) | 25 ± 3.0 | 34 ± 3.0 | 42 ± 8.0 | 22 ± 2.0 | 33 ± 2.0 | 46 ± 6.0 |

**S2: Drug loading and release**

**Figure S20**: Doxorubicin (Doxo) molecule (ChemBioDraw and Mercury).

**Table S8**: Doxo characteristics.

| Drug | MW (g/mol) | λ_ex_ (nm) | λ_em_ (nm) | Size (nm) |
| --- | --- | --- | --- | --- |
| Doxo | 543.52 | 470 | 560 | 1.5 |

**Quantification of Doxo by fluorescence measurements**: Fluorescence characterization in solution was performed using a UV-VIS Cary300 spectrometer. The amount of Doxo loaded into CS was measured indirectly by measuring the Doxo fluorescence ($\frac{\lambda_{ex}}{\lambda_{em}}=\frac{470}{560}$) remaining in the supernatants:

(H_2_O [from the first wash of the composite in the in-situ methodology] and MeOH [from the first wash of the composite in the after-synthesis methodology]) after centrifugation and washing of the composite. The Doxo concentration was determined by interpolation of the measured fluorescence intensity (I) to a previously constructed calibration curve with 6 different concentrations from 0.5 to 5 μg/mL (**Figure S21**).

**Figure S21:** Calibration curve of Doxo in H_2_O and MeOH was obtained from fluorescence under excitation at 520 nm, applying the Beer-Lambert law. Fluorescence intensity (I) at the maximum emission peak as a function of Doxo concentration c is plotted, and the calibration equation is obtained by fitting a linear regression line to collected data; R^2^ is the coefficient of determination.

The encapsulation efficiency (EE%) of Doxo was calculated using **Equation (S1)**, while the loading efficiency (LE%) was determined using **Equation (S2)**.

$EE\%= \frac{amount of drug released (mg)}{amount of drug encapsulated (mg)}\times100$ ***Equation S1***

$LE\%= \frac{amount of drug loaded (mg)}{amount of NC (mg)}\times100$ ***Equation S2***

**Table S9**: Conditions of Doxo encapsulation in-situ.

| Drug | [Drug] (μg/mL) | [Zn^2+^] (M) | V_Zn2+_ (μL) | Total volume (mL) | Solvent |
| --- | --- | --- | --- | --- | --- |
| Doxo | 150 | 0.25 | 100 | 1 | H_2_O |
| Doxo | 250 | 0.25 | 100 | 1 | H_2_O |
| Doxo | 500 | 0.25 | 100 | 1 | H_2_O |

**Table S10**: Conditions of Doxo encapsulation post-synthesis.

| Drug | [Drug] (μg/mL) | [CS] (nM) | V_Doxo_ (mL) | Total volume (mL) | Solvent |
| --- | --- | --- | --- | --- | --- |
| Doxo | 150 | 0.1 | 1 | 1 | MeOH |
| Doxo | 250 | 0.1 | 1 | 1 | MeOH |
| Doxo | 500 | 0.1 | 1 | 1 | MeOH |

**Figure S22**: Shape tunning when using different amounts of Doxo: TEM showing the effect of Doxo encapsulated by the 1^st^ method (in-situ encapsulation): a) 150 μg/mL; b) 250 μg/mL; and c) 500 μg/mL and by the 2^nd^ method (post-synthesis encapsulation): d) 150 μg/mL; e) 250 μg/mL and f) 500 μg/mL.

**Figure S23**: BETSI analysis for CS composite.

**Figure S24**: BETSI analysis for CS-Doxo(a).

**Figure S25**: BETSI analysis for CS-Doxo(i).

| Sample | S_BET_ (m^2^/g) |
| --- | --- |
| ZIF-8 | 1601 |
| CS | 1452 |
| CS-Doxo(*a*) | 1395 |
| CS-Doxo(*i*) | 754 |

**Table S11**: S_BET_ values for ZIF-8, CS, CS-Doxo(a) and CS-Doxo(i).

**Figure S26**: Degradation over time by XRD of (A) CS-Doxo(i)-PMA and (B) CS-Doxo(a)-PMA over a period of three months in water.

**Figure S27**: Study of stability over time for CS-Doxo(i)-PMA before and after freeze drying process, by DLS (A) and Zeta-potential (B). St.Dev. refers to the error between the measurements.

**Figure S28**: TEM images of the CS-Doxo(i)-PMA in different conditions: NaOH (0.1M), DMEM, PBS pH = 7.5, and PBS pH 4.5, over 24h of incubation.

**Figure S29**: (A) Comparison of the heating performance of Fe_3_O_4_@ZIF-8 (CS) composite in water, viscous media, and DMEM at 50% (v/v); (B) ACHLs recorded at f = 110 kHz and H = 24 kA/m.

**S3: Cell studies – Supplementary figures**

**Table S12**: Concentration of Zn used for the toxicity test and the corresponding Doxo concentrations.

| Zn concentration (mg/L) | Doxo (*i*) concentration (μg/mL) |
| --- | --- |
| 1 | 0.014 |
| 2 | 0.028 |
| 4 | 0.056 |
| 8 | 0.112 |

**Figure S30**: Cell viability of MDA-MB-468 cells after 24, 48, and 72 h of incubation with the range of concentrations of CS-PMA (n≥3) (A) and CS-Doxo(i)-PMA (n≥3) (B).

**Figure S31:** Uptake of CS-Doxo(i)-PMA composite after 2, 24, 48 h of incubation in U-87 MG cell line. (A) U-87 MG cell singlets exhibiting Doxo fluorescence in % of total cell singlets population after 2, 24, 48 h of incubation with 2 mgZn/L CS-Doxo(i)-PMA (n=3); (B) example of the raw data recorded by flow cytometry for one of the experiments showing in green the cells positive to Doxo; (C) mean fluorescence values recorded for all the cell singlets incubated with 2 mgZn/L CS-Doxo(i)-PMA divided by the mean fluorescence values recorded for all the cell singlets of the corresponding controls; (D) The raw data of one of the experiments showing the shift of the fluorescence peak between 2 and 24 h of incubation.

**Figure S32**: Excitation/emission wavelength positions of Hoechst 33342 (blue), Doxo (green), LysoTracker Deep Red (red).

*i=0*

*while (i<=11){*

*roiManager("Select", i);*

*//Setting the counter, with i<= N, N-number of ROI selected -1*

*run("Duplicate...", "title=A duplicate");*

*run("Clear Outside");*

*run("Select None");*

*//Renaming the file to "A" to be able to run the further actions, preparing the files*

*run("Split Channels");*

*close();*

*selectWindow("C1-A");*

*close();*

*//Selecting the needed fluorescence channels "C2" and "C3"*

*run("Smooth");*

*//using mean filters with different radii*

*selectWindow("C3-A");*

*run("Smooth");*

*setAutoThreshold("Default dark");*

*setThreshold(33, 255);*

*setOption("BlackBackground", true);*

*//Selecting the threshold values for channel 3. To select the threshold values we must first choose these values manually by applying the "adjust - threshold" command and seeing which values give us the closest selection of fluorescence signal*

*run("Convert to Mask");*

*//Creating a mask based on fluorescence that will be overlayed on the mask of another fluorescence channel*

*selectWindow("C2-A");*

*run("Duplicate...", " ");*

*setThreshold(27, 255);*

*run("Measure");*

*//Selecting the threshold values for channel 2. To select the threshold values we must first choose these values manually by applying the "adjust - threshold" command and seeing which values give us the closest selection of fluorescence signal*

*selectWindow("C2-A");*

*imageCalculator("Subtract create", "C2-A","C3-A");*

*selectWindow("Result of C2-A");*

*setThreshold(27, 255);*

*run("Measure");*

*// The first measurement result will give us the total value of the DOXO-associated fluorescence on the image, the second value will represent the DOXO-associated fluorescence outside of the LysoTracker mask*

*close();*

*selectWindow("C2-A");*

*close();*

*selectWindow("C2-A-1");*

*close();*

*selectWindow("C3-A");*

*close();*

*i=i+1;}*

*roiManager("Delete");*

*run("Select None");*

**Figure S33**: Macros written for ImageJ program in order to quantify the colocalization of the composite (channel 2 fluorescence) and lysosomes (channel 3 fluorescence). Explanations are inserted in green and are started with “//” symbols to avoid their interference in the code running.

**Figure S34**: (A) Confocal images of the unstained and untreated U-87 MG cells exhibiting no fluorescence in any channels. (B) Confocal images of U-87 MG cells incubated with CS-Doxo(i)-PMA for 24, 48, and 72 h. Merged image of all channels; channel 3 (LysoTracker Deep Red, approx. 668 nm emission); channel 2 (Doxo, false colour, approx. 590 nm emission); channel 1 (Hoechst 33342, approx. 461 nm emission). All the images are recorded with the same laser and microscope configuration parameters; no modifications are made to the images. Scale bar: 20 μm.

*run("Duplicate...", "title=A duplicate");*

*//Renaming the file to "A" to be able to run the further actions, preparing the files*

*setBackgroundColor(0, 0, 0);*

*run("Clear Outside");*

*//Preparing the image for the analysis, removing the background behind the ROI selection done manually*

*run("Split Channels");*

*close();*

*selectImage("C1-A");*

*close();*

*//Leaving only the fluorescence channel that will be analyzed; in this case it is channel 2 – lysotracker-associated fluorescence, but it can be modified to select channel 1 – DOXO-related fluorescence.*

*run("In [+]");*

*run("In [+]");*

*run("Smooth", "stack");*

*//using mean filters with different radii*

*setAutoThreshold("Default no-reset");*

*setThreshold(75, 255, "raw");*

*//Selecting the threshold values for channel 2. To select the threshold values we must first choose these values manually by applying the "adjust - threshold" command and seeing which values give us the closest selection of fluorescence signal*

*run("Measure");*

*close();*

**Figure S35**: Macros written for ImageJ program. Explanations are inserted in green and are started with “//” symbols to avoid their interference in the code running.

**Figure S36**: Corresponding viability test of Doxo at different concentrations corresponding to the loading efficiency of Doxo(i). Viability was assessed by MTT assay. Values are presented as mean with error bars indicating the standard deviation (SD) for n=3 independent experiments.

**Figure S37**: MDA-MB-468 cell viability in Ctrl – complete DMEM; 2 mgZn/L CS-PMA, 2 mgZn/L CS-Doxo(i)-PMA. Incubated for 1.5 h - “Incubated”; exposed to RT for 1.5 h – “RT”; exposed to 1 h of AMF – “AMF-1 h”; exposed to 1.5 h of AMF – “AMF-1.5 h” (n≥3). Statistical analysis: Brown-Forsythe and Welch ANOVA tests. Dunnett T3 multiple comparison correction; P value reporting style is NEJM: 0.12 (ns); 0.033 (*); 0.002 (**); < 0.001 (***).

**S4: Experimental section**

**Materials**

All chemicals were obtained from commercial sources and used without further purification. Iron(III) acetylacetonate (Fe(acac)_3_, 99%) and decanoic acid (DA, 99%) were purchased from Acros. Iron(II) chloride tetrahydrate (FeCl_2_.4H_2_O), iron(III) chloride hexahydrate (FeCl_3_.6H_2_O), hexadecyltrimethylammonium bromide (CTAB, 98%), 2-methylimidazole (MeIM, 99%), zinc nitrate hexahydrate (Zn(NO_3_)_2_, 98%), poly(isobutylene-alt-maleic anhydride) (PMA, Mw=~6000, 85%), sodium oleate (97%), dodecylamine (DDA, 98%), oleic acid (OA, 90%), 1-octadecene (90%), dibenzylether (DBE, 98%), acetone (99.5%), chloroform (CHCl_3_, 99.8%), tetrahydrofuran (THF, 99.9%), hexane (95%), methanol (MeOH, 99.8%), ethanol (EtOH, 99.8%), toluene (99.7%) and isopropanol (99.8%) were purchased from Sigma Aldrich. Doxorubicin hydrochloride (Doxo, Aldrich, European Pharmacopeia Reference Standard). Squalane (SQ, 98%) was purchased from Alfa Aesar, and Milli-Q water filtered through 0.22 μm pore size hydrophilic filters (18.2 MΩ cm) was supplied by Milli-Q® Integral Water purification System. All chemicals were used as received.

**Elemental analysis**

Induced coupled plasma optical emission spectroscopy (ICP-OES) analysis was performed on an ICAP 6000 spectrometer (Thermo Scientific) to quantify the concentration of the elements of interest. For the Fe_3_O_4_ and Fe_3_O_4_@CTAB NPs, 10 μL samples were digested in 1 mL of aqua regia (HCl:HNO_3_ ratio 3:1 (v/v)) overnight and then diluted with 10 mL of Milli-Q water prior to the measurement. In the case of the ZIF-8, CS, and CS-PMA composites, 5 or 10 μL samples were digested in 2 mL of HNO_3_:H_2_O_2_ ratio 1:1 (v/v) for 15 min at 180 °C in a CHEM MARS6 oven and diluted with 10 mL of Milli-Q water before the measurement.

**Transmission electron microscopy (TEM)**

Overview transmission electron microscopy (TEM) analyses were carried out on JOEL-JEM-1400 electron microscope, equipped with a W thermionic electron source and a 11Mp Orius CCD Camera (Gatan company, USA), with an acceleration voltage of 100 kV. The samples were prepared by drop-casting a droplet of the sample solution onto a 400 mesh ultra-thin carbon-coated copper grid, with a subsequent removal of the solvent by evaporation at RT. STEM characterization was done in a Thermo Fisher Spectra 300 operated at 300 kV; chemical maps were acquired using a Dual-X detector and processed using Velox.

**Scanning electron microscopy (SEM)**

The morphology and size of the nanoparticles were investigated with SEM. Images were acquired with a FESEM Zeiss Ultra Plus operated at 3 kV or 20 kV. The InLens detector was utilized for image acquisition.

**Dynamic light scattering (DLS) and Zeta potential (ZP)**

Dynamic light scattering (DLS) and the zeta potential (ZP) were recorded by means of a Malvern NanoZS instrument equipped with a 4.0 mW HeNe laser (633 nm). An equilibration time of 1 min was allowed prior to each reading, and at least three replicate measurements were made for each sample.

**PXRD Diffraction Analysis**

Powder X-ray diffraction (PXRD) patterns were obtained using a PANalytical Empyrean X-ray diffractometer equipped with a 1.8 kW Cu Kα ceramic X-ray tube and a PIXcel3D 2 × 2 area detector operating at 45 kV and 40 mA. The diffraction patterns were collected in air at RT using parallel-beam geometry and symmetric reflection mode. The samples were examined in the range of 2θ between 5° and 80°. All PXRD samples were prepared by drop-casting a concentrated solution on a zero-diffraction quartz wafer. PXRD patterns were elaborated and compared with reference patterns using Highscore 4.1 software by Panalytical.

**Magnetic characterization**

Magnetic measurements were performed on a superconducting quantum interface device (SQUID) from Quantum Design Inc. Samples were prepared by dropping 100 μL (0.5 mgFe/mL in chloroform or methanol) of samples onto 100 mg of Teflon film and left to dry before the analysis. Hysteresis loops were recorded within the magnetic field of ±4800 kA/m at 5 K and 298 K. Temperature-dependent magnetization, zero field cooled (ZFC), and field cooled (FC) curves were recorded under a magnetic field of 4 kA/m. In a ZFC protocol, the sample was cooled from room temperature to 5 K without any magnetic field, and the magnetization was measured during heating from 5 – 400 K at 2 K/min under the magnetic field of 4 kA/m. In an FC protocol, samples were cooled down to 5 K under the 4kA/m magnetic field, and the magnetization was recorded during heating mode from 5 – 400 K under the same heating rate. The results were normalized by the metal mass obtained by ICP (Fe in the case of Fe_3_O_4_ and Fe or Fe+Zn in the case of CS).

**AC magnetic measurements**

AC hysteresis loops of magnetic samples made from Fe_3_O_4_@CTAB, and CS-PMA were measured using an AC magnetometer device (AC Hyster advance, Nanotech Solutions). Samples of desired concentration (1 mgFe/mL diluted in water) and volume (40 µL) were inserted in a capillary tube (diameter 3 mm, length 76 mm) and analyzed under dynamic magnetic field conditions of frequency 110, 200, and 300 kHz and field range 16 – 24 kA/m.

**Raman spectroscopy**

Raman spectra were collected using a Renishaw in Via Raman Microscope. Experiments were conducted in backscattering geometry at RT, employing a 785 nm laser excitation wavelength. The laser beam, set at 5% power, was focused on the sample using a 50x objective for a 10s exposure time to prevent overheating or sample transformations. All spectra were recorded over the wavenumber range of 100 - 2000 cm^-1^ with 5 scans accumulated to reduce spectral noise. Raman peak deconvolution was performed using Origin 2018.

**N_2_ adsorption-desorption analysis**

N_2_ sorption isotherms at 77 K were measured using a Micromeritics 3Flex Adsorption Analyzer. Approximately 20 mg of each sample was activated overnight under high vacuum at 90 °C prior to analysis. Data analysis was performed using the 3Flex V5.03 software (Micromeritics Instrument Corp., Norcross, GA, USA). Braunauer-Emmet-Teller (BET) surface area values were determined using BETSI software in alignment with previously reported values.[1]

**Thermogravimetric analysis (TGA)**

TGA measurements were performed using a TA Instruments Inc. SDT Q-600 thermobalance. The samples were heated under nitrogen from 25 to 800 °C at a rate of 5 °C/min under an air flow of 25 mL/min. Prior to analysis, all samples were lyophilized.

**UV-VIS/Fluorescence**

Fluorescence spectra of the samples were measured using UV-VIS spectroscopy. Spectra for samples in both MeOH and H_2_O were acquired over the range of 400 – 700 nm using a Varian Cary Eclipse spectrometer with 1 cm quartz cells (QS 10 x 4 mm light path).

**Synthesis of materials**

***Synthesis of IONPs (spherical shape)***

Iron oxide spherical nanoparticles with a diameter of 22 nm were synthesized in two steps, following a published procedure by Ritchie Chen et al.[2] Briefly, the metal-oleate precursor was prepared by reacting sodium oleate (160 mmol) with the metal chloride salts, FeCl_3_ (40 mmol) and FeCl_2_ (20 mmol) in 100 mL ethanol, 100 mL MiliQ water, and 200 mL hexane, and heating to reflux at 60 °C for 4 h. After removal of the aqueous phase, the organic phase was heated to 70 °C for 2 h and then placed under vacuum at 110 °C for an additional 2 h to remove residual solvent, leaving behind a viscous metal-oleate product. Next, to obtain the 22 nm IONPs, in a 250 mL 3-necked flask, the metal-oleate complex (5 mmol) and oleic acid (2.5 mmol) were dissolved in 25 mL of 1-octadecene and evacuated for 30 min. Then the solution was heated up to 200 °C under N_2_ flow, further heated to 325 °C at a rate of 1 °C/min, and held at the specific temperature for 1 h. After the heating mantle was removed and the solution was cooled to RT, the entire reaction solution was transferred to a 50 mL conical tube along with 1:1 mixture of ethanol and hexane. The sample was then centrifuged twice at 6000 rpm for 10 min and finally redispersed in toluene.

***Synthesis of IONPs (cubic shape)***

Iron oxide nanocubes with a diameter of 18 nm were synthesized following a published procedure with minor modifications.[3] Briefly, in a 100 mL three-necked flask, 0.353 g (1 mmol) of iron(III) acetylacetonate, with 1.033 g (6 mmol) of decanoic acid, and dissolved in 13 mL of dibenzyl ether (DBE) and 12 mL of Squalane. The resulting deep red solution was degassed at 65 °C, reaching 15-20 μbar after 1 h. After switching to N_2_, the mixture was heated up to 200 °C (5 °C/min) and kept for 2 h. The temperature was then increased to 300 °C at a heating rate of 7 °C/min and kept for 1 h. The nitrogen flow rate was fixed at intermediate flow (40 - 80 bubbles per min). After cooling down to RT, the black colloidal solution was washed twice with an excess amount of acetone (45 mL) and centrifuged at 4500 rpm for 20 min to collect the particles as a black precipitate. Finally, the collected particles were kept in 10 mL of chloroform for further experiments.

***Coating of IONPs (spherical and cubic shapes) with CTAB***

IONPs having oleic acid or decanoic acid on the surface were coated with CTAB with a simple procedure, in which the IONPs volume and concentration were adapted for each sample. The CTAB amount was accordingly adjusted to ensure a consistent surface coverage of 1000 CTAB molecules per nm^2^. As an example, in the case of IONPs in cubic shape, the procedure consists of mixing 150 μL (172 nM of NPs concentration) of IONPs with 1 mL CTAB (0.05 M), corresponding to a ratio of 1000 CTAB molecules per nm^2^. The mixture was placed in a sonicator bath and kept for 1 h at 60 °C. The deep black solution was then cooled down to RT and washed by means of centrifugation at 10000 rcf for 10 min. The supernatant was discarded, and the dark pellet was redispersed in a CTAB solution (0.16 mg/mL) and washed again. Finally, the pellet was redispersed in CTAB (0.16 mg/mL) and centrifuged again at 3000 rcf for 3 min. Both pellet and supernatant were kept in 1 mL CTAB (0.16 mg/mL) for further experiments.

***Synthesis of pristine ZIF-8 nanoparticles***

The synthesis of pristine ZIF-8 nanoparticles was adapted from a published procedure with minor modifications.[4] 1 mL of MeIM (1.3 M) aqueous solution was placed in a glass vial under sonication at RT. Then, 1 mL of Zn(NO_3_)_2_·6H_2_O (0.025 M) aqueous solution was added, and immediately after, 1 mL of CTAB (0.2, 0.4, or 0.6 mg/mL) aqueous solution was added. The mixture was left for 10 min sonicating and left undisturbed for 3 h at RT, observing during this time the gradual appearance of whitish turbidity, which indicated the formation of the ZIF-8 particles. Finally, the particles were collected by centrifugation at 7000 rcf for 10 min, washed twice with methanol (MeOH), and finally redispersed in 1 mL of MeOH.

***Synthesis of CS composite***

The synthetic protocol for the CS composites was equivalent to the one previously described for pristine ZIF-8 particles, with the substitution of a CTAB-coated IONPs solution (Fe_3_O_4_@CTAB, 4 nM of NPs concentration) containing 0.2, 0.4, or 0.6 mg/mL CTAB instead of the CTAB solution. In this case, the appearance of greyish turbidity was gradually observed during the formation of ZIF-8. The resulting nanoparticles were redispersed in 1 mL of MeOH.

***Scale-up synthesis of CS composites***

All reagents, meaning MeIM (1.3 M), Zn(NO_3_)_2_ (0.025 M)_,_ and Fe_3_O_4_@CTAB solutions (4 nM of NPs concentration), were prepared manually in 15 mL falcons, to a total volume of 7 mL, and placed in the designated place in the robot deck. The 24 mL glass vials were used as reactor pots and placed in a 24-well polypropylene rack. The automated procedure was initiated by orbital shaking at 500 rpm. Then, the robot started to dispense 1 mL of MeIM in each of the 24 (8 mL) glass vials at an addition rate of 400 μL/s. When all vials were filled, it started to dispense in one row at the time, the zinc solution (400 μL/s) and right after the nanoparticle’s solution (400 μL/s) followed by 5 min shaking. The liquid dispensing process was programmed and automatically carried out. After the shaking step, the robot procedure was stopped, and the vials were left at RT for 3 h.

***Synthesis of PMA-based amphiphilic polymer (poly[isobutylene–alt-maleic anhydride]-graft-dodecyl]***

The synthesis of the PMA was performed as described previously with minor modifications.[4,5] In a 250 mL round-bottom flask was placed 3.084 g of poly(isobutylene-alt-maleic anhydride (20 mmol, Mw ~ 6000 g/mol of a whole polymer, molecular weight of one PMA monomer unit M_PMA_~154 g/mol), 2.7 g of dodecylamine (DDA, 20 mmol), and 130 mL of THF. The mixture was left under stirring at 60 °C in a preheated mantle, overnight. After this time, the THF was evaporated in the rotavapor (70 rpm, 40 °C). The resulting sponge-shaped product was diluted in 15 mL CHCl_3_, which was sonicated until fully dispersion and concentrated to 5 mL in the rotavapor. After this, 100 mL of hexane was added and sonicated a bit. The resulting solution was divided into 3 falcons and placed in the fridge (-20 °C) for 3h. Finally, the white product was recovered by means of centrifugation at 7000 rcf for 3 min, dried under vacuum, and redispersed in CHCl_3_ to obtain a final concentration of 0.05M.

***Phase transfer of the CS composites in water with PMA-based amphiphilic polymer***

The composites, dispersed in MeOH were mixed with PMA (0.05M) in CHCl_3_ solution, with the PMA amount adjusted according to the NPs concentration and diameter to provide a surface coverage equivalent to 150 monomers per nm^2^ surface area of composite (assuming a spherical particle of the different diameters obtained). As an example, 851 μL of CS (1.1 gZn/L, 0.11 nM of NPs concentration) with a d_h,N_ of 238 nm was mixed with 200 μL PMA (0.05 M), in a 50 mL round-bottom flask, and the solvent (3:1 MeOH:CHCl_3_) was slowly evaporated in a rotary evaporator. Then, the dried product was suspended in 500 μL of NaOH (0.1 M) in a 1.5 mL Eppendorf tube, aided by sonication. The resulting nanoparticles were collected by centrifugation at 7000 rcf for 10 min, washed with NaOH (0.01 M), and finally kept concentrated in 50 μL of NaOH (0.01 M).

***In-situ Doxo encapsulation within the ZIF-8***

The synthesis protocol was similar to the synthesis of the CS composite, but adding the desired amount of Doxo (e.g., 150 mg) in the Zn(NO_3_)_2_ solution, always keeping the concentration of 0.025 M for the Zn(NO_3_)_2_, and the total volume of 1 mL. As an example, 100 μL Zn(NO_3_)_2_ (0.25M) was mixed with 30 μL Doxo (5000 μg/mL) and 870 μL H_2_O in a 1.5 mL Eppendorf tube. Then, the 1 mL solution was added over 1 mL MeIM (1.3 M) under sonication. Finally, 1 mL CTAB-coated NPs (4 nM of NPs concentration) was added, and the mixture was left for 10 min sonicating and left undisturbed for 3 h at RT, observing during this time the gradual appearance of whitish turbidity, which indicated the formation of the ZIF-8 particles. Finally, the particles were collected by centrifugation at 7000 rcf for 10 min, washed twice with methanol (MeOH), and finally redispersed in 1 mL of MeOH.

***Post-synthesis Doxo encapsulation within the ZIF-8***

The encapsulation protocol consists of adding 1 mL of the desired amount of Doxo (e.g., 150 mg) in MeOH over the pre-synthesized CS. The particles were then washed with MeOH by centrifugation at 7000 rcf for 10 min, keeping the supernatant for analysis.

***Stability test***

The stability test of the Doxo-loaded composites was determined in water by DLS, TEM, and XRD. Therefore, prior to the test, the composites (either prepared by *in-situ* or after-synthesis) were transferred to water using the PMA. The water stability of CS-Doxo(*i*)-PMA and CS-Doxo(*a*)-PMA was studied over 3 months by repeating measurements of hydrodynamic size (d_h_) by DLS, complemented by TEM imaging and XRD analysis.

To ensure long-term stability, samples were freeze-dried immediately after preparation using a lyophilizer Lio 5P and stored in this lyophilized state until further use. Prior to characterization or application, the samples were redispersed in water.

***Doxo release study in vitro***

The stability of the CS-Doxo*(i)*-PMA was studied in 2 sets of CS solutions, such as PBS pH 4.5 mimicking the pH of the lysosomes, and Dulbecco’s Modified Eagle’s Medium (DMEM, high glucose) supplemented with 10% fetal bovine serum (heat-inactivated FBS), 2% penicillin−streptomycin (10000 U/mL), and 1% L-glutamine (200 mM) (further referred to as complete DMEM). The supernatants (100 μL) were collected at different time points (30 min, 2 h, 6 h, 14 h, 23 h, and 24 h), centrifuged once (7000 rcf for 10 min), and analyzed by TEM. The CS were resuspended with 100 μL of fresh solutions to maintain the same volume of 1 mL.

To quantify the amount of non-specific and controlled release of Doxo from our CS-Doxo(*i*)-PMA, the DMEM solution was chosen for the study. The CS solutions were exposed to either 37 °C or AMF (*f* = 265 kHz, H = 12 kA/m) treatments, respectively. First, five sets of solutions (1 mL, at 15 mgZn/L) were placed in an incubator at 37 °C and under stirring (150 rpm) for 24 h. The supernatants (100 μL) were collected at different time points (30 min, 2 h, 6 h, 14 h, and 24 h), centrifuged once (7000 rcf for 10 min), and analyzed by fluorescence. The percentage release was calculated by using **Equation S3**.

$Release Doxo \left( \% \right)=\frac{release Doxo at time t}{total amount of Doxo loaded}\times100$ ***Equation S3***

Another 2 sets of solutions (1 mL, at 15 mgZn/L) were in the same condition in the incubator at 37 °C and under stirring (150 rpm) for 6 h and 24 h. After this time, they were subjected to AMF at fixed conditions of H = 12 kA/m and *f* = 265 kHz for 1.5 h (3 cycles of 30 min) using a NanoTherics device. At the end of every release 1 h period, the CSs were centrifuged once (7000 rcf, 10 min), and the supernatant was analyzed by fluorescence while the pellet was redispersed and analyzed by TEM.

**Cell culture**

All the cell culture reagents were purchased from Gibco. U-87 MG human glioblastoma and MDA-MB-468 breast cancer cell lines were purchased from ATCC. Cells were cultured in attachment in Dulbecco’s Modified Eagle’s Medium (DMEM, high glucose) supplemented with 10% fetal bovine serum (heat-inactivated FBS), 2% penicillin−streptomycin (10000 U/mL), and 1% L-glutamine (200 mM) (further referred to as complete DMEM) at 37 °C, 5% CO_2_, and 95% relative humidity. All experiments were performed at cell passage numbers between 10 and 30.

***In vitro cytotoxicity studies***

For the *in-vitro* Cytotoxicity Assay, U-87 MG cells were cultured and grown overnight in 2D and plated in 96-multiwell plates at a cell density of 8x10^3^ cells per well, while the MDA-MB-468 at 1.5x10^4^ cells per well in order to reach confluency at the end of the test (72 h) but not to exceed it. The cells were incubated with CS-PMA, and CS-Doxo(*i*)-PMA at different concentrations in DMEM (1, 2, 4, and 8 mgZn/L) for 24, 48, and 72h. Control cells did not receive treatment with NPs, but the DMEM was changed at the same time as the other cells were receiving NPs. At the corresponding time points, an MTT cytotoxicity assay was conducted. For this 10 μL of 5 mg/mL Thiazolyl Blue Tetrazolium Bromide in PBS was added to each well to make 10% of the total volume in each well and left for incubation for 2 h. After the incubation of MTT with cells, the formed crystals were dissolved in 100 μL DMSO per well. Optical density was read at 570 nm with background subtraction at 630 - 690 nm in a Cary 50 spectrometer. The results are plotted after at least three independent experiments are performed. The mean values and error bars, representing standard deviation, are calculated between the experiments. The values for each condition are calculated based on the corresponding control values, as follows using **Equation S4**:

$cell viability \left( \% \right)=\frac{I_{S}}{I_{C}}\times100$	**Equation S4**

where I_S_ is the final intensity values for the samples, and I_C_ is the final intensity values for the control non-treated cells.

***Analysis of composite uptake by flow cytometry***

U-87 MG cells were plated in 24-multiwell plates at a cell density of 1x10^5^ per well in complete DMEM and grown for 24 h at 37 °C. The cells were incubated with 500 μL of CS-Doxo*(i)*-PMA composite at 2 mgZn/L solution for 2, 24, and 48 h at 37 °C. The cells were washed three times with 0.5% Bovine Serum Albumin (BSA) in PBS (phosphate-buffered saline) to remove the CS not internalized by cells. Next, the cells were trypsinized with 200 μL trypsin per well, collected into individual 2 mL tubes, and centrifuged at 1000 rpm for 5 min. The resulting pellets were then resuspended in 400 μL of PBS and placed on ice. The Doxo signal was recorded using the 488 nm excitation laser and phycoerythrin (PE) emission filter 582/15 nm filter.

***Subcellular Localization of the composite***

To localize the CS-Doxo(*i*)-PMA composite in cells, 1x10^5^ U-87 MG cells were seeded in the 3 of the 4-chamber Cellview culture dishes (Greiner BioOne), while in the remaining one, only cells were seeded, which is used for the control and kept in culture for 24h. After this time, 500 μL of 2 mgZn/L of CS-Doxo(*i*)-PMA composite was added to the cell monolayer and incubated for 24, 48, and 72 h. After the incubation, and 20 minutes before confocal microscopy the complete DMEM in wells was replaced with 500 μL of 10 nM solution of LysoTracker Deep Red (647 nm excitation, Invitrogen) in complete DMEM, which was then replaced with 500 μL of 10 nM solution of Hoechst 33342 Fluorescent Stain (350 nm excitation, Invitrogen) in complete DMEM. The control cells remained unstained and untreated to set up the confocal parameters, avoiding any signals in the control cells. After obtaining the z-stacks with Zeiss LSM780 confocal microscope, all the files were analyzed with ImageJ software comparing the location of the Doxo-associated fluorescence and LysoTracker-associated fluorescence. Excitation/emission wavelength positions of Hoechst 33342 (blue), Doxo (green), and LysoTracker Deep Red (red) are shown in **Figure S32**. For each condition, more than 130 individual cells were analyzed. All the procedures done in ImageJ are described and recorded in **Figure S33**, SI.

***Real-time observation of Doxo release upon AMF application***

3.5x10^5^ U-87 MG cells were seeded in a Cellview culture dish (Greiner BioOne) and kept in culture for 24 h. U-87 MG cells were incubated with 2 mL of 2 mgZn/L solution of CS-Doxo*(i)*-PMA composite in complete DMEM for 24 h. 20 minutes prior to confocal microscopy, the complete DMEM in wells was replaced with 2000 μL of 10 nM solution of LysoTracker Deep Red (647 nm excitation, Invitrogen) in complete DMEM and further replaced with 3000 μL of complete DMEM. Finally, the cells were exposed to AMF at fixed conditions of H = 16 kA/m and *f* = 300 kHz for 1 h under the confocal microscope (Zeiss LSM 780) using a homemade miniaturized electromagnet.[6] The parameters of the magnetic field applied were selected to keep the treatment conditions below the clinical limits. The increase in volume accounts for the possible evaporation of the medium while at the microscope. The images with/without AMF were obtained with LSM780 confocal microscope (Zeiss) every 3 minutes for 1 h in total. The quantification of lysotracker and Doxo fluorescence was done through ImageJ Macro Language Programmer cell by cell (**Figure S35**, SI). For each condition, the number of individual cells analyzed ranged from 50 to 120. The error bar on the graphs is the standard error between all cell measurements for each time point and each parameter.

***Cell proliferation after AMF application***

To confirm the effect of the AMF application on the cell’s proliferation, the viability assay was carried out. The experiment was performed as follows: 1x10^5^ U-87 MG cells and 1.5x10^5^ MDA-MB-468 cells in 500 μL of complete DMEM were seeded per well in a 4-cell view chamber Cellview culture dishes (Greiner BioOne) and kept in culture for 24 h to ensure their attachment. 500 μL of fresh medium for the control cells and 500 μL of CS-PMA and CS-Doxo(*i*)-PMA at 2 mgZn/L in complete DMEM were then added to the corresponding wells. After 24 h of incubation, all samples were washed with fresh DMEM, filled with 700 μL of it, and the four identical 4-cell view chambers were subjected to:

1) 60 minutes of AMF (*f* = 262.6 kHz, H = 19.8 kA/m).
2) 90 minutes of AMF (*f* = 262.6 kHz, H = 19.8 kA/m).
3) 90 minutes of RT exposure (aprox. 17 °C).
4) 90 minutes of incubation at 37 °C, 5 % CO_2_, and 95% relative humidity

The AMF was applied on the NanoTherics magneTherm^TM^ device at fixed conditions of *f* = 263 kHz, and H= 20 kA/m. After the corresponding treatment chambers are placed back in the incubator and left for another 24 h. Presto Blue (Invitrogen) cell viability analysis was done the day after the AMF application. For that, 400 μL complete DMEM containing 10% of stock PrestoBlue™ Cell Viability Reagent was added to each chamber and incubated for 1.5 h in case of U-87 MG cells, and 45 min in case of MDA-MB-468 cells. The resulting solution was transferred to 96-well plates with 100 μL per well and the absorbance was analyzed at 570 nm and 600 nm with normalization by subtraction. The results were plotted considering the control sample left in the incubator as 100% viability. The error bars are calculated between the corresponding results of four independent experiments.

***Statistical analysis***

The cytotoxicity and cell proliferation experiments were obtained from at least three independent experiments (n≥3). Statistical significance for the experiments comparing multiple groups passing the normality test was determined using Brown-Forsythe and Welch ANOVA tests. For the calculated fluorescence intensity signals of Doxo and lysotracker changing with time (**Figure 8F,H**), Kruskal-Wallis and Dunn’s multiple comparison test for nonparametric data were applied. For the overall trend comparison of the calculated fluorescence intensity signals of Doxo and lysotracker Mann-Whitney nonparametric test was applied (**Figure 8I**). For all the statistical analyses, the confidence interval is 95% and the P value reporting style is NEJM: 0.12 (ns); 0.033 (*); 0.002 (**); < 0.001 (***).

**S5: References**

1.	Cedrún-Morales M, Ceballos M, Soprano E, et al. Light-Responsive Nanoantennas Integrated into Nanoscale Metal–Organic Frameworks for Photothermal Drug Delivery. Small Sci. 2024;4(8):2400088. doi:10.1002/smsc.202400088

2.	Chen R, Christiansen MG, Anikeeva P. Maximizing Hysteretic Losses in Magnetic Ferrite Nanoparticles via Model-Driven Synthesis and Materials Optimization. ACS Nano. 2013 Oct 22;7(10):8990–9000. doi:10.1021/nn4035266

3.	Silvestri N, Gavilán H, Guardia P, et al. Di- and tri-component spinel ferrite nanocubes: synthesis and their comparative characterization for theranostic applications. Nanoscale. 2021;13(32):13665–80. doi:10.1039/D1NR01044A

4.	Carrillo-Carrión C, Martínez R, Navarro Poupard MF, et al. Aqueous Stable Gold Nanostar/ZIF-8 Nanocomposites for Light-Triggered Release of Active Cargo Inside Living Cells. Angew Chem Int Ed. 2019;58(21):7078–82. doi:10.1002/anie.201902817

5.	Hühn J, Carrillo-Carrion C, Soliman MG, et al. Correction to Selected Standard Protocols for the Synthesis, Phase Transfer, and Characterization of Inorganic Colloidal Nanoparticles. Chem Mater. 2021 Jun 22;33(12):4830–4830. doi:10.1021/acs.chemmater.1c01764

6.	Connord V, Clerc P, Hallali N, et al. Real-Time Analysis of Magnetic Hyperthermia Experiments on Living Cells under a Confocal Microscope. Small. 2015;11(20):2437–45. doi:10.1002/smll.201402669
